# A systematic review of the applications of artificial intelligence and machine learning in autoimmune diseases

**DOI:** 10.1038/s41746-020-0229-3

**Published:** 2020-03-09

**Authors:** I. S. Stafford, M. Kellermann, E. Mossotto, R. M. Beattie, B. D. MacArthur, S. Ennis

**Affiliations:** 1 Department of Human Genetics and Genomic Medicine, University of Southampton, Southampton, UK; 2 Institute for Life Sciences, University of Southampton, Southampton, UK; 3 Department of Paediatric Gastroenterology, Southampton Children’s Hospital, Southampton, UK

**Keywords:** Autoimmune diseases, Machine learning, Predictive medicine

## Abstract

Autoimmune diseases are chronic, multifactorial conditions. Through machine learning (ML), a branch of the wider field of artificial intelligence, it is possible to extract patterns within patient data, and exploit these patterns to predict patient outcomes for improved clinical management. Here, we surveyed the use of ML methods to address clinical problems in autoimmune disease. A systematic review was conducted using MEDLINE, embase and computers and applied sciences complete databases. Relevant papers included “machine learning” or “artificial intelligence” and the autoimmune diseases search term(s) in their title, abstract or key words. Exclusion criteria: studies not written in English, no real human patient data included, publication prior to 2001, studies that were not peer reviewed, non-autoimmune disease comorbidity research and review papers. 169 (of 702) studies met the criteria for inclusion. Support vector machines and random forests were the most popular ML methods used. ML models using data on multiple sclerosis, rheumatoid arthritis and inflammatory bowel disease were most common. A small proportion of studies (7.7% or 13/169) combined different data types in the modelling process. Cross-validation, combined with a separate testing set for more robust model evaluation occurred in 8.3% of papers (14/169). The field may benefit from adopting a best practice of validation, cross-validation and independent testing of ML models. Many models achieved good predictive results in simple scenarios (e.g. classification of cases and controls). Progression to more complex predictive models may be achievable in future through integration of multiple data types.

## Introduction

### Autoimmune disease

**Fig. 1:**
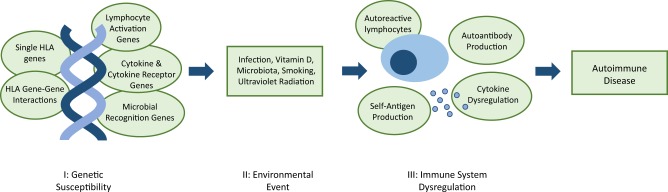
The three factors contributing to autoimmune disease development. **I** genetic susceptibility is conferred by a combination of genes that may include genes encoding human leukocyte antigen (HLA) innate and adaptive immune proteins, and directly or indirectly affect the regulation of the immune system. **II** examples of potential environmental triggers for dysregulation. **III** autoantibody production alone will not always result in disease development; self-antigen production and subsequent elevated immune response is necessary.^3^

Three elements contribute to autoimmune disease development: genetic predisposition, environmental factors and immune system dysregulation (Fig. 1). Due to the heterogeneity of onset and progression, diagnosis and prognosis for autoimmune disease is unpredictable.

A predisposition to autoimmunity is strongly linked to genetics, and caused by defects in mechanisms that result in loss of self-tolerance.^1^ Autoimmune disease develops after further immune system dysregulation, in both the innate and adaptive immune system.^2^ Microbial antigens, foreign antigens and cytokine dysregulation, can cause induction of self-reactive lymphocytes.^3^ Moreover, hyper-activation of T and B cells may occur, along with a change in the duration and quality of their response, which further disrupts the homeostasis of the immune system.^2^

The prevalence of autoimmune disease is difficult to estimate; diseases are variably represented across different studies and no definitive list exists.^4–6^ There is a reported prevalence rate of between 4.5%^5^ and 9.4%,^4^ across all autoimmune diseases.

### The importance of personalised medicine

Personalised care is valuable for autoimmune disease, with variability within the disorders,^7^ and presence of autoimmune comorbidities for 15–29% of patients.^8–11^ Arguably, patients with multiple autoimmune comorbidities would particularly benefit from personalised healthcare for the causal molecular mechanism as opposed to specialist treatment of symptoms.

### The data revolution

Standard patient care generates diverse clinical data types. Examples of such data include laboratory test results from blood or urinary samples, symptoms at diagnosis and images obtained using colonoscopies and magnetic resonance imaging (MRI). The majority of these data are reproduced longitudinally over a chronic disease course.

In addition to this wealth of clinical data, ‘omic data—such as patients’ genomic, transcriptomic and proteomic profiles—are now increasingly available. ‘Omic data are large, as molecular measurements are made on a genome-wide scale,^12^ and high throughput omics technologies have allowed fast analysis of these data. The inclusion of multiple types of ‘omic data into machine learning models may give a more complete picture of autoimmune disease, leading to novel insights.

### The need for artificial intelligence and machine learning

Combined clinical and ‘omic data have limited utility without methods for interpretation. Artificial intelligence and machine learning techniques have the capacity to identify clinically relevant patterns amongst an abundance of information,^13^ fulfilling an unmet need. The ability to stratify patient’s using these data has implications for their care, from estimation of autoimmune disease risk, diagnosis, initial and ongoing management, monitoring, treatment response and outcome.

### Defining artificial intelligence and machine learning

The terms “machine learning” (ML) and “artificial intelligence” (AI) are often conflated. Artificial intelligence is the study of methods to imitate intelligent human behaviour (for example to make decisions under conditions of uncertainty). Machine learning is a subset of AI that focuses on the study of algorithms that enable a computer to perform specific tasks (typically classification or regression) without specific instructions, but instead inferring patterns from data.^14^ Both AI and ML differ from traditional statistical methods as they focus on prediction and classification from high-dimensional data, rather than inference. Successful ML requires robust data from which it can learn. These data must be sufficiently abundant to enable the model to be robust and generalisable to unseen data.

### Supervised and unsupervised machine learning

Two types of ML are discussed here: supervised and unsupervised learning. During supervised learning, an algorithm is trained on a “training dataset” to recognise the patterns that are associated with specific “labels” (for example, healthy or diseased). Once predictive patterns have been learned from training data, the ML algorithm is then able to assign labels to unseen “test data”. In a well-trained model, the patterns identified in the training data will generalise to the test data. Brief descriptions of some of the most common supervised ML techniques referred to in this review are summarised in Box 1.

For unsupervised learning, training data are unlabelled, and the algorithm instead attempts to find and represent patterns within the data, for example by identifying clusters based upon the similarity of the examples. Other types of ML exist, but are reviewed elsewhere.^15^ Some of the more common unsupervised methods discussed in this review include hierarchical clustering and self-organising maps.

Box 1 Popular supervised machine learning methods Neural networks: outputs are learned from inputs via a series of nested nonlinear functions, encoded in a network of “neurons”, which may vary in its topology.^59^

Decision trees: outputs are learned from inputs via a series of yes/no questions that successively divide the predictor space into discrete piece.^175^

Random forest: a simple ensemble method that grows a large number of decision trees, each of which see only a subset of the data, and learns output from input by combining the predictions.^79^

k nearest neighbours: learns output from input by comparing the identity of each data point to its (k) nearest neighbours.^117^

Support vector machine: a binary classification method that can be adapted to multiclass classification or regression. They seek to partition the predictor space into two, such that data points from each class are concentrated on one side of the decision boundary.^118^

Natural language processing: a set of advanced ML methods that seek to extract sentiment from text.^19^

### The pros and cons of alternative machine learning models

Recommendations cannot be made on the best model to use in general, as this is always situation specific and dependent on data type, size and dimensionality. Decision trees are simple and highly interpretable, but they rarely achieve performance accuracies higher than other algorithms. Using the random forest method can improve performance, at the cost of losing some interpretability. K nearest neighbours is a non-parametric method, and copes well when complex boundaries separate classes, but this flexibility can lead to poor classification results due to overfitting.^16^ Neural networks and support vector machines have similar strengths and weakness: they achieve high accuracies, and can extract linear combinations of features, but interpretability is poor, scaling to very large data can be difficult, and they are not robust to outliers.^17^

Technical aspects regarding the operation and fitting of machine learning algorithms are outside the scope of this systematic review, but comprehensively discussed elsewhere.^16,17^

### Avoiding overfitting

**Fig. 2:**
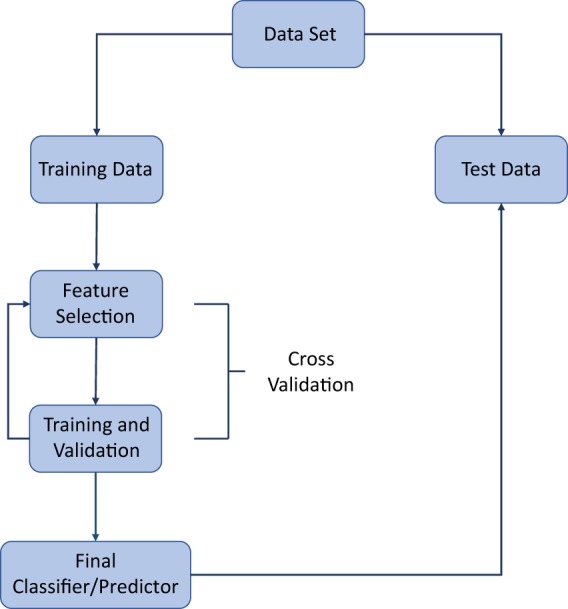
Simplified workflow for developing a machine learning model. This includes the cycle of feature selection, training and validation that is required to avoid overfitting (cross validation).

Machine learning models are often complicated, and can involve optimizing many free parameters. For this reason, they are prone to overfitting. Overfitting is the process by which the algorithm learns patterns that are specific to the training data but do not generalise to test data. For example, there may be some random technical error in the training data that is not of clinical relevance, yet is learned by the algorithm. Training any model accurately while avoiding overfitting is a central part of an ML pipeline. If data are abundant and/or the ML model is computationally expensive to train then the standard strategy is to remove a portion of the data for training, optimize the model on the remaining portion, and finally determine the model performance by comparison with the unseen test portion. If data are not abundant, a process known as cross-validation is typically employed (Fig. 2). There are many variations of cross-validation but they are all essentially generalisations of the training/test splitting process described above. For example, in k-fold cross validation, the data are randomly split into k subsets, with all but one subset used to train an ML model, and the remaining subset used to test the model. This process leads to the generation of *k* ML models. Each subset of the data is used only once as a test set, and overall model performance is determined by averaging the performance of the k models (Box 2 describes model evaluation metrics).^18^

Box 2 Metrics for ML method evaluation Accuracy: percentage of correct predictions.^198^

Area under the receiver-operator curve (AUC): appropriate for binary classification problems, this method uses a plot of sensitivity versus specificity to determine model performance.^16^

Balanced accuracy: measure of the total number of correct predictions in either class, therefore taking into account an unbalanced dataset.^198^

F-score: an accuracy measure calculated using precision and recall.^199^

Out-of-bag error: this metric applies to tree-based ensemble methods, and measures the test error by comparing predictions with true labels for samples that were not used in the construction of a particular decision tree.^16^

Precision: equivalent to positive predictive value.^16^

Recall: another term for sensitivity.^16^

*R*^*2*^: measures the amount of variation explained by the model regression.^16^

Sensitivity: correctly identified true positives.^16^

### Artificial intelligence, machine learning and autoimmune disease

This systematic review aims to inform on the current status of the application of artificial intelligence and machine learning methods to autoimmune disease to improve patient care. To the best knowledge of the researchers, this is the first study on this topic. The review identifies the most common methods, data and applications, the issues surrounding this exciting interdisciplinary approach, and promising future possibilities.

## Results

### Summary of results

**Fig. 3:**
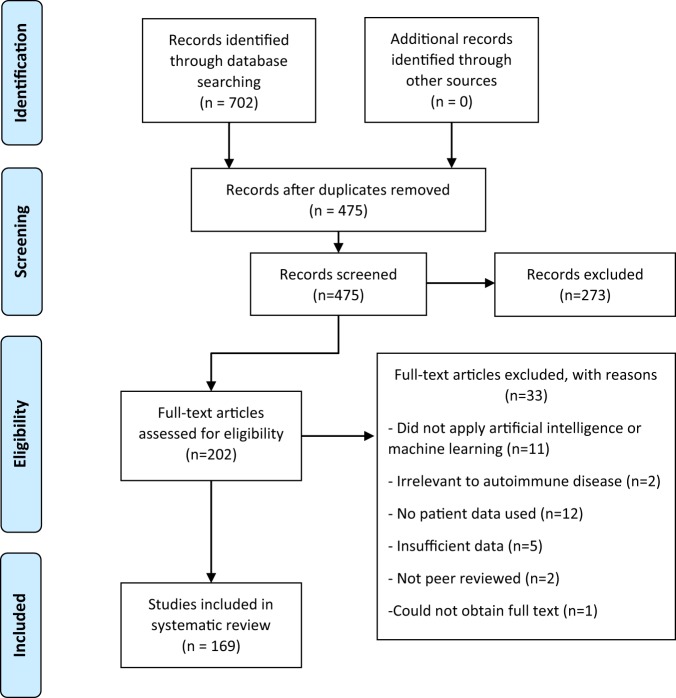
Methodological flowchart and number of papers reviewed at each stage. The inclusion and exclusion criteria are applied to the title and abstract at the screening step and to the full article at the eligibility step. During the screening step, it was unclear from some abstracts if the article fulfilled the criteria, and therefore a full read is completed at the eligibility step to clarify the status of those records. Two reviewers completed screening independently, and where consensus could not be reached, a third reviewer assessed these articles and decided whether they were included or excluded.

Of 702 papers identified in database searches, 169 were selected for inclusion in the analysis, 227 duplicates were removed, 273 records were excluded based on the abstract and 33 were excluded after reading the full article (Fig. 3) using the criteria described above. A summary and detailed information for qualifying studies are described in Table 1 and Supplementary Table 1, respectively. Six diseases included in the database search returned no studies that met the inclusion and exclusion criteria (Addison disease, myasthenia gravis, polymyalgia rheumatica, Sjӧgren syndrome, systemic vasculitis and uveitis).

**Table 1:** Machine learning and artificial intelligence applications to autoimmune diseases.

Disease	Number of studies	Years	Most popular classification/prediction application(s)	Most popular machine learning method(s)	Median sample size (min, max)	Data types used
Multiple sclerosis	41^30,45,50,51,60,61,71,91–93,100,101,111,117–144^	2008–2019	Diagnosis, Prognosis, Disease Subtype	Type of Regression, Random Forest, Support Vector Machine	99 (12, 12566)	Clinical, Survey, Genetic, MRI, Lipid Markers, SNPs, Gait Data, Immune repertoire, Gene Expression
Rheumatoid arthritis	32^20–22,26,27,31,32,40–42,46–48,52,59,62–64,70,72,80–82,88,97,145–151^	2003–2018	Risk, Diagnosis, Early Diagnosis, Identify Patients	Support Vector Machine, Variations of Random Forest, Neural Network and Decision Tree	338 (22, 922199)	Medical Database, Immunoassay, Metagenomic, Microbiome, GWAS/SNP, Clinical, Movement Data, Amino acid analytes, Transcriptomic, EMRs, Ultrasound images, Proteomic, Laser images
Inflammatory bowel disease	30^33–36,43,57,69,73,79,83–86,94,95,98,152–165^	2007–2018	Diagnosis, Response to Treatment, Disease Risk, Disease Severity	Random Forest, Support Vector Machine	273 (50, 53279)	Clinical, Colonoscopy Images, Metagenomic, Gene Expression, GWAS, Microbiota, miRNA Expression, EMRs, Exome, MRI
Type 1 diabetes	17^37–39,67,68,102–104,166–174^	2009–2018	Disease Management	Novel Methods/Hybrid Models, Neural Network, Support Vector Regression	23 (10, 10579)	Clinical, Red Blood Cell Images, VOCs, GWAS/SNPs
Systemic lupus erythematosus	14^19,23,44,49,89,96,175–182^	2009–2018	Variations of prognosis, Diagnosis	Logistic Regression, Neural Network, Random Forest Decision Tree	318 (14, 17057)	Clinical, Electronic Health Records, Drug Treatment, SNPs, MRI, Exome, Gene Expression, Proteomic, Urine Biomarkers
Psoriasis	11^53,74–77,99,112,183–186^	2007–2018	Diagnosis, Disease Severity	Support Vector Machine	540 (80, 22181)	Digital Image, GWAS, Proteomic, RNA Biomarkers
Coeliac disease	7^24,25,54,65,66,78,187^	2011–2018	Diagnosis	Random Forest, Logistic Regression, Bayesian Classifier, Support Vector Machine, Logistic Model, Natural Language Processing, Combined Fuzzy Cognitive Map and Possibilistic Fuzzy c-means clustering.	465 (47, 1498)	VOCs, Clinical, Peptide, EMRs
Thyroid diseases	6^188–193^	2008–2018	Diagnosis	Hybrid Models	215 (215, 7200)	Clinical
Autoimmune liver diseases	5^58,87,90,194,195^	2009–2018	Prognosis	Variations on Random Forest	288 (64, 787)	Clinical, Clinical Trial, Microbiome
Systemic sclerosis	4^55,113,196,197^	2016–2018	Diagnosis, Treatment, Prognosis	Support Vector Machine, Random Forest	119 (37, 991)	Gene Expression, Nailfold capillaroscopy images, Peripheral Blood Mononuclear cell data (flow cytometry, DNA, mRNA)

Information includes the number of studies per autoimmune disease, the years they occurred, popular applications and methods and data types used. Median sample size was a better representation than mean, due to large cohorts in studies using data from genome-wide association studies and electronic medical records.

*EMR* electronic medical record, *GWAS* genome-wide association study, *miRNA* micro RNA, *MRI* magnetic resonance imaging, *SNP* single nucleotide polymorphism, *VOC* volatile organic compound.

Machine learning and artificial intelligence are most commonly applied to multiple sclerosis (MS), rheumatoid arthritis (RA) and inflammatory bowel disease (IBD). MS, IBD and RA models used the most types of data, including 13 studies generating models using two data types (always including clinical data). Random forests and support vector machines were the most commonly used methods throughout diseases and applications. Clinical data were used in models for every type of autoimmune disease, and models using genetic data were created for the majority of disorders. The variety in methodological approaches, applications and data, as well as use of validation methods (Supplementary Table 1) renders meta-analysis of these methods inappropriate.

The applications for ML can be categorised into six broad topics: patient identification, risk prediction, diagnosis, disease subtype classification, disease progression and outcome and monitoring and management.

### Identification of patients

Studies utilised ML methods to identify patients with autoimmune diseases from electronic medical records,^19–25^ and employed natural language processing. Gronsbell et al. worked to improve the efficiency of algorithms for this purpose.^26,27^ These algorithms are intended to replace International Classification of Diseases billing codes, which have error rates of between 17.1–76.9% due to inconsistent terminology.^19^ Electronic medical records also identified comorbidities associated with alopecia and vitiligo using natural language processing. This identified similar autoimmune comorbidities for both diseases.^28,29^

### Identifying and assessing autoimmune disease risk

Prediction of disease risk^30–39^ and identification of novel risk factors through feature selection^40–44^ was documented for IBD, type 1 diabetes (T1D), RA, systemic lupus erythematosus (SLE) and MS. Fifteen studies employed genetic data, using either sequencing arrays (GWAS) or exome data (nine studies), individual SNPs^38^ within in the HLA regions^37,45^ or pre-selected genes,^41^ or gene expression data.^30,43^ Only one study employed clinical data,^31^ and two others combined clinical and genomic data.^30,45^ Popular models included random forest, support vector machine and logistic regression.

### Diagnosis

Patient diagnosis was the most frequent ML application, and this approach was used for all autoimmune diseases. Distinguishing cases from healthy controls was an aim for 27 studies. Diagnostic classification models used patients with other autoimmune diseases as controls,^46–49^ differentiated between diseases with overlapping or similar symptoms or phenotype, for example stratifying coeliac disease and irritable bowel syndrome,^50–56^ or examined classification of multiple autoimmune diseases.^57,58^ ML specifically for early diagnosis was specified by seven studies for the later onset degenerative conditions MS and RA.^48,59–64^ Other diagnostic applications included distinguishing coeliac disease from an at-risk group^65,66^ and differentiating those who have complications in T1D.^67,68^ Random forests and support vector machine most frequently utilised.

### Classifying disease subtypes

Disease subtypes in one RA, two IBD, and six MS studies were classified by ML. Three types of unsupervised clustering were used by these studies: hierarchical clustering for identifying novel IBD subtypes;^69^ consensus clustering to identify high, low and mixed levels of inflammation in RA;^70^ and agglomerative hierarchical clustering to cluster MS by genetic signature.^71^ Two of these studies employed support vector machine,^69,70^ which is a popular supervised method in general, as well as random forest. There was wide variation in data types used. These included clinical (in particular MRI), genetic, RNA sequencing and gene expression data.

### Disease progression and outcome

Disease progression and outcome was a focus for 27 studies. Other considered issues were disease severity^72–78^ in psoriasis, RA, IBD and coeliac disease; treatment response^79–87^ in IBD, RA and primary biliary cirrhosis (PBC); and survival prediction^88–90^ in PBC, RA and SLE. Other models focused on improved image segmentation to aid prognoses^91–96^ for IBD and MS. Disease progression and outcome was the second-most prevalent area for model development. Throughout, the most common models were support vector machines, random forest and neural networks. The majority of data used was clinical, with very few papers utilising ‘omic data.^86,97–99^

### Monitoring and management

Ten different studies of type 1 diabetes (T1D) used ML for monitoring and management: four predicted blood glucose level, four identified or predicted hypoglycaemic events, and two supported decision making using case-based reasoning or decision support systems. The majority of models used clinical data. Three models were developed using activity measurements for monitoring movement in MS, and one in RA. Support vector regression was used most frequently.^100–104^

## Discussion

### Validation and independent testing

Eighteen studies only used hold-out validation, not including studies with random forest models, where cross validation is unnecessary, or neural networks, where this process can be too computationally intensive. Eleven studies did not use any validation method, and so model integrity and applicability is unconfirmed. Methods that use hold-out validation have the potential to provide useful information, but it is accepted that unless the dataset is very large, these models are not as robustly validated as those that have used k-fold or leave-one-out cross validation, or a combination of cross-validation and testing on an independent dataset.

Only 14 of 169 studies combined cross-validation with independent test data for evaluating their models. These studies did not have any model types or applications in common. Clinical and genomic data were most common inputs for these studies. Models that used cross-validation and independent test data were applied to a number of the autoimmune diseases.

The research reviewed here demonstrates that, much like the disease studied, the ML models and methods used are heterogeneous. It can be difficult then, to determine which methods should be taken forward to clinical application. Alternatively, models from existing studies could be combined. Models have utilised different types of ‘omic data, including proteomic, metagenomic and exome data. More popular has been sequencing array (SNP/GWAS) data, particularly when predicting autoimmune disease risk. By far the most prevalent type of data is the use of clinical and laboratory data.

To optimise the use of these data types, accessibility is key, and EMRs allow easy extraction of these data. Some researchers have moved beyond only storing medical data in these systems. The eMERGE (electronic medical records and genomics) network combines the genomic and EMR repositories to further genomic medicine research.^105^ Other studies such as SPOKE (Scalable Precision Medicine Oriented Knowledge Engine), wish to integrate these data within the storage platform, by building a knowledge network using unsupervised machine learning that informs on how data types such as GWAS, gene ontology, pathways and drug data are connected to EMRs.^106^ Improving knowledge of how these data are related is a key step towards implementing precision medicine.

Many models were created for autoimmune disease diagnosis, more specifically classifying those with disease and controls. The majority achieved high classifier performance (where any combination of the following metrics are over these thresholds: accuracy > 81%, AUC > 0.95, Sensitivity > 82, Specificity > 84), and provided evidence of machine learning’s utility in diagnostics.

Identifying the molecular diagnosis to inform tailored treatment strategies has revolutionised cancer prognoses, improving patient outcomes and quality of life, along with economic benefits to the treatment provider. Targeted therapies such as monoclonal antibodies and small molecule inhibitors transformed treatment of some cancers, or improved patient survival times.^107^ Key to precision treatment has been the identification of the driver mutations specific to the cancer type.^108^ Machine learning has been utilised for cancer classification^109,110^ and discovery of relevant pathways.^109^ Across the spectrum of autoimmune diseases, there has traditionally been a one-size-fits-all approach to patient therapeutics. The expectation is that machine learning represents a necessary key tool that will use ‘big’ data to stratify patients and move towards personalised treatment approaches that have proven so effective in cancer. Proof of this concept has already been demonstrated through machine learning to stratify patient’s inflammation status in RA,^70^ and further investigate IBD subtypes.^69^

Six models from the evaluated studies returned more than one of the following measures as either 1 or 100%: AUC, accuracy, precision and recall, sensitivity and specificity.^59,67,68,111–113^ This perfect performance indicates that a model may not be required, as there exists data that classifies the groups without error. An alternative explanation of apparently optimal performance may reside in poor implementation of cross-validation strategies.

Common metrics reported are accuracy, AUC, and sensitivity and specificity. However, accuracy is inferior to AUC, particularly when imbalanced datasets are used.^114^ The AUC measure is unaffected by imbalanced data, but precision-recall curves may reflect model performance more accurately.^115^ Dataset rebalancing methods should potentially be utilised more for a thorough review of model performance.

When creating and evaluating a model, increasing focus could be placed on which measure is more important, sensitivity or specificity. Scully et al. demonstrated this, where a lesion segmentation model could achieve high specificity (99.9%) through labelling all tissue as non-lesion.^96^

An ML model by Ahmed et al.^62^ provides evidence for using an additional independent test dataset subsequent to cross validation. In their study, the AUC dropped by 0.25, indicating decreased model performance on new data.

Studies included in this systematic review have shown that artificial intelligence and machine learning models provide useful insight, despite the heterogeneity of presentation, diagnosis, disease course and patient outcome. However, the heterogeneity in data used, models and model evaluation cause difficulties in obtaining consensus. Furthermore, the number of autoimmune diseases this literature search focussed on was restricted, and may have resulted in an incomplete picture of ML applied to autoimmune diseases.

From this analysis, it seems appropriate to advocate for standardised methods of model evaluation, by utilising a combination of cross validation and independent test data for model validation. Increased confidence in model results allows for more complex model creation, by layering data types or combining classifiers. These models could be applied to more difficult tasks that reflect the complexity of autoimmune disease. With these advances, AI and ML have the potential to bring personalised medicine closer for patients with complex and chronic disease.

## Methods

### Autoimmune disease selection

Autoimmune diseases selected for the systematic review are based on prevalence estimates^4^ and include Addison disease, alopecia, Coeliac disease, Crohn’s disease, ulcerative colitis, type 1 diabetes, autoimmune liver diseases, hyper- and hypo-thyroidism, multiple sclerosis, myasthenia gravis, polymyalgia rheumatica, psoriasis, psoriatic arthritis, rheumatoid arthritis, Sjӧgren syndrome, systemic sclerosis, systemic lupus erythematosus, systemic vasculitis, uveitis and vitiligo.

### Literature search

The literature search was performed electronically with OvidSP using MEDLINE from 1946, and EMBASE from 1974. A search was also performed on the Computers & Applied Sciences Complete database available on EBSCO. The literature search was completed in December 2018. All searches conformed to the same structure: the words “machine learning” or “artificial intelligence” combined with the chosen search term(s) for each autoimmune disease (see Table 2). Boolean operators OR and AND (for combining search terms) were used in order to streamline the procedure. In both databases, the title, abstract and subject terms/keyword headings assigned by authors were searched (last search 17/12/2018).

**Table 2:** Search terms used in OvidSP and EBSCO for each autoimmune disease.

Autoimmune disease	Disease Search Term(s) Used
Addison’s disease	Addison*
Alopecia	Alopecia
Celiac disease	Celiac, Coeliac
Inflammatory bowel disease	Inflammatory bowel disease, Crohn* disease, ulcerative colitis
Type 1 diabetes	Type 1 Diabetes, Insulin dependent Diabetes^?^
Autoimmune hepatitis	Autoimmune hepatitis, chronic active hepatitis, primary biliary cirrhosis, primary sclerosing cholangitis
Thyroid disease	Autoimmune thyroiditis, Hashimoto* thyroiditis, Hashimoto* disease, Grave* disease, hyperthyroid*, hypothyroid*
Multiple sclerosis	Multiple sclerosis
Myasthenia gravis	Myasthenia gravis
Polymyalgia rheumatica	Polymyalgia rheumatica
Psoriasis	Psoriasis
Psoriatic arthritis	Psoriatic arthritis
Rheumatoid arthritis	Rheumatoid Arthritis
Sjӧgren syndrome	Sjogren syndrome
Systemic sclerosis	Systemic sclerosis
Systemic lupus erythematosus	Lupus
Systemic vasculitis	Polyarteritis nodosa, microscopic polyangiitis, granulomatosis with polyangiitis, eosinophilic granulomatosis with polyangiitis.
Uveitis (iridocyclitis)	Uvetitis, iridocyclitis
Vitiligo	Vitiligo

Asterisk (*) and question mark (?) are wildcard characters used for searching the databases OvidSP and EBSCO.

### Inclusion and exclusion criteria

Studies that applied ML methods to autoimmune diseases listed above, or to complications that arise from autoimmune diseases were included. Studies not written in English, published prior to 2001, that did not use real human patient data, were not peer reviewed, or were review papers were also excluded. This systematic review conforms to the Preferred Reporting Items for Systematic Reviews and Meta-Analyses (PRISMA) standards.^116^

### Reporting summary

Further information on research design is available in the Nature Research Reporting Summary linked to this article.

## Supplementary information

Supplementary Information

Reporting Summary

## Data Availability

The data (papers) that support the findings of this study are available publicly. Full list of records identified through database searching are available on reasonable request from the authors.
