# Supplementary material for: A systematic review of the applications of artificial intelligence and machine learning in autoimmune diseases
Source: NPJ Digit Med. 2020 Mar 9;3:30. doi: 10.1038/s41746-020-0229-3 (PMC7062883; doi:10.1038/s41746-020-0229-3)
Supplement: Supplementary file 1 — Supplementary Information [file 41746_2020_229_MOESM1_ESM.pdf]

Supplementary Material for A Systematic Review of the Applications of Artificial Intelligence and Machine Learning to Autoimmune Diseases.

Supplementary Table 1 Detailed information for each study included in the systematic review, grouped by autoimmune disease.
AA=Alopecia Areata, ACPA = Anti-Citrullinated Peptide Antibodies, AI = Renal Pathology Acute Index, AID = Autoimmune Disease, AUC = Area under the ROC Curve, axSpA = Axial Spondyloarthritis, CeD = Coeliac Disease, CFS = Chronic Fatigue Syndrome, CGM = Continuous Glucose Monitoring, CI = Renal Pathology Chronic Index, CIS = Clinically Isolated Syndrome, COPD = Chronic Obstructive Pulmonary Disease, CrD = Crohn's Disease, D-IBS = Diarrhoea-Predominant Irritable Bowel Syndrome, EDSS = Expanded Disability Status Scale, EHR = Electronic Health Record, EMR = Electronic Medical Record, FP = False Positive, GWAS = Genome Wide Association Study, HC = Healthy Controls, IBD = Inflammatory Bowel Disease, LASSO = Least Absolute Shrinkage and Selection Operator, LDA = Linear Discriminant Analysis, LH-PCR = Length Heterogeneity Profile or Fingerprint, ME = Myalgic Encephalomyelitis, MF = Mycosis Fungoides, MFI = Motor Function Impaired, MFP = Motor Function Preserved, MLP = Multilayer Perceptron, MRI = Magnetic Resonance Imaging, MS = Multiple Sclerosis, OA = Osteoarthritis, OND = Other neurological diseases, P = Psoriasis, PAFS = Psoriasis and Psoriatic Arthritis Follow-up Study, PAPS = Primary Antiphospholipid Syndrome, PPMS = Primary Progressive Multiple Sclerosis, PRMS = Progressive Relapsing Multiple Sclerosis, PsA = Psoriatic Arthritis, PsC = Cutaneous-only Psoriasis, PSC = Primary Sclerosing Cholangitis, PsV = Psoriasis Vulgaris, RA = Rheumatoid Arthritis, RBC = Red Blood Cell, RF = Random Forest, RSME = Root Mean Square Error, RRMS = Relapsing Remitting Multiple Sclerosis, SLE = Systemic Lupus Erythematosus, SNP = Single Nucleotide Polymorphism, SpA = Spondyloarthropathy, SPMS = Secondary Progressive Multiple Sclerosis, SSc = Systemic Sclerosis, SVM = Support Vector Machine, T1D = Type 1 Diabetes, T2D = Type 2 Diabetes, UC = Ulcerative Colitis, VOC = Volatile Organic Compound.

Paper	Multiple AIDs Studied	Prediction or Classification Task	ML Type	Machine Learning Method	Study Size (N)	Type of Data	Best Results (Metrics) Reported from validation or cross-validation, and where conducted, the test set.	Cross-Validation
**Multiple Sclerosis**								
Briggs et al. 2019 [1]	No	Disease Progression	Supervised	Multivariable Regression	N=1515	Clinical, Survey and Genetic Data	.	10-fold cross-validation
Ahmadi et al. 2019 [2]	No	Diagnosis	Supervised	Neural Network	N=12 (n(MS)=5, n(HC)=7)	Clinical Data	Colour task: Accuracy=91%, Sensitivity=83%, Specificity=96%.<br>Direction Task: Accuracy=90%, Sensitivity=82%, Specificity=96%.	Leave-one-out cross-validation
Zhang et al. 2019 [3]	No	Disease Progression	Supervised	Random Forest	N=84	MRI Data	Shape Based: AUC=0·85, Sensitivity=0·94, Specificity=0·5.<br>Shape based with lesion segmentation tool: AUC=0·82, Sensitivity=0·95, Specificity=0·33	3-fold cross-validation
Zurita et al. 2018 [4]	No	Diagnosis	Supervised	Support Vector Machine	N=150 (n(RRMS)=104, n(HC)=46)	MRI Data	RRMS vs HC: Accuracy=87·8%, Precision=89·7%, Sensitivity=88%, Specificity=87·6%.<br>RRMS (EDSS > 1·5) vs HC: Accuracy=88·6%, Precision=91·6%, Sensitivity=87·5%, Specificity=89·8%.	10-fold cross-validation
Wang et al. 2018 [5]	No	Diagnosis	Supervised	Neural Network	N=1357 (n(MS)=676, n(HC)=681) images.<br>N=64 (n(MS)=38, n(HC)=26) patients	MRI Data	Accuracy=98·77, Precision=98·75, Sensitivity=98·77%, Specificity=98·76%	Hold-out validation
Neeb et al. 2018 [6]	No	Diagnosis	Supervised	k Nearest Neighbours	N=97 (n(MS)=52, n(HC)=45)	MRI Data	Data not affected by motion: False prediction rate=16·3%.<br>All data: False prediction rate=25·5%	Leave-one-out cross-validation

Paper	Multiple AIDs Studied	Prediction or Classification Task	ML Type	Machine Learning Method	Study Size (N)	Type of Data	Best Results (Metrics) Reported from validation or cross-validation, and where conducted, the test set.	Cross-Validation
Lotsch et al. 2018 [7]	No	Diagnosis	Supervised and Unsupervised	Emergent self-organising maps, Random Forest	N=403 (n(MS)=102, n(HC)=301)	Lipid Marker Data	ESOM balanced accuracy=98%.<br>Random forest: AUC=100%, Area under the precision recall curve=98·87%, Balanced accuracy=100%, Sensitivity=100%, Specificity=100%	Nested cross-validation
Tacchella et al. 2017 [8]	No	Disease Progression	Supervised	Random Forest/Human Rating Hybrid	N=84	Clinical Data	AUC=0·725 (180 days), 0·694 (360 days), 0·696 (720 days)	Leave-one-out cross-validation
Lopez et al. 2018 [9]	No	Disease Subtype	Unsupervised	Agglomerative hierarchical clustering algorithm	N=191	SNP Data	Rand Index=0·96	10-fold cross-validation
Supratak et al. 2018 [10]	No	Risk of Disease	Supervised	Support Vector Regression	N=32	Gait Speed Data	R-value=0·98	. (Individual models)
Sacca et al. 2018 [11]	No	Early Diagnosis	Supervised	Random Forest or Support Vector Machine	N=37 (n(RRMS)=18, n(HC)=19)	MRI Data	Accuracy=85·7%, Sensitivity=100%, Specificity=66·7% (SVM and RF)	5-fold cross-validation
Mowry et al. 2018 [12]	No	Risk of Disease	Supervised	Logistic Regression	N=6552 (n(MS)=3276, n(HC)=3276)	Clinical/Survey and Genetic (HLA) Data	.	10-fold cross-validation (tuning parameter only)
Yoo et al. 2018 [13]	No	Early Diagnosis	Supervised and Unsupervised	Deep Learning, LASSO and Random Forest	N=99 (n(RRMS)=55, n(HC)=44)	MRI Data	AUC=88·0% Accuracy=87·9% Sensitivity=87·3%, Specificity=88·6%	11-fold cross-validation
Kiiski et al. 2018 [14]	No	Disease Progression	Supervised	Machine Learning approach with Penalised Linear Regression	N=78 (n(MS)=35 (22 RRMS, 13 SPMS), n(HC)=43)	Clinical Data	Cognitive functioning: r-value 0·35 (baseline), 0·44 (13 months).<br>Processing Speed and Working Memory: r-value 0·27 (baseline), 0·39 (13 months)	10-fold cross validation, nested cross validation
Fiorini et al. 2015 [15]	No	Disease Subtype	Supervised	Ordinary Least Squares Regression or Regularised Least Squares Regression	N=457 (n(RRMS)=170, n(SPMS)=205, n(PPMS)=68, n(PRMS)=8, n(Benign)=6)	Clinical Scales, Patient Reported Outcomes (anthropometric and questionnaires) Data.	Accuracy=78·32 (Ordinary least squares), 78·24 (regularised least squares), F1 score=0·701 (Ordinary least squares), 0·702 (regularised least squares)	Hold-out validation, testing set
Zhong et al. 2017 [16]	No	Disease Progression	Supervised	Support Vector Machine	N=72 (n(MFP)=26, n(MFI)=25, n(HC)=21)	MRI Data	HC vs MFI: AUC=0·9448, Accuracy=88·34%, Sensitivity=96·00%, Specificity=85·71%.<br>HC vs MFP: AUC=0·8416, Accuracy=84·16%, Sensitivity=88·46%, Specificity=85·71%.<br>MFP vs MFI: AUC=0·8338, Accuracy=85·61%, Sensitivity=92%, Specificity=84·62%.	Leave-one-out cross-validation

Paper	Multiple AIDs Studied	Prediction or Classification Task	ML Type	Machine Learning Method	Study Size (N)	Type of Data	Best Results (Metrics) Reported from validation or cross-validation, and where conducted, the test set.	Cross-Validation
Lotsch et al. 2017 [17]	No	Diagnosis	Unsupervised	Emergent self-organising feature maps	N=403 (n(MS)=102, n(HC)=301)	Clinical (Lipid Serum) Data	Balanced Accuracy=94·6%, Sensitivity=89·2%, Specificity=100%	.
Karaca et al. 2017 [18]	No	Disease Subtype	Supervised	Convex Infinite Kernel Approach (CIKA)	N=139 (n(MS)=120, n(HC)19)	MRI and EDSS Data	Accuracy=0·8889	10-fold cross-validation
Ostmeyer et al. 2017 [19]	No	Diagnosis	Supervised	Logistic Regression Model	N=125 (n(train)=71 RRMS + 12 OND; n(val)=60 RRMS + 42 OND)	Clinical (Immune Repertoire) Data	Cross-validation: Accuracy=87%<br>Independent Test Data: AUC=0·75, Accuracy=72%	Leave-one-out cross-validation, independent test data
McGinnis et al. 2017 [20]	No	Disease Progression	Supervised	Support Vector Regression	N=47	Gait Measurement Data	RMSE 0·14m/s	Leave-one-subject-out cross-validation
Zhao et al. 2017 [21]	No	Disease Progression	Supervised	Support Vector Machine	N=1693	Clinical and MRI Data	G0: Accuracy=0·67, Sensitivity=0·81, Specificity=0·59.<br>G1: Accuracy=0·68, Sensitivity=0·82, Specificity=0·58.<br>G2: Accuracy=0·65, Sensitivity=0·80, Specificity=0·57.<br>G3: Accuracy=0·54, Sensitivity=0·52, Specificity=0·55.	10-fold cross-validation
Ion-Margineanu et al. 2017 [22]	No	Disease Subtype	Supervised	Linear Discriminant Analysis, Random Forest or Support Vector Machine	N=105 (n(MS)=87, n(HC)=18)	Clinical and MRI Data	CIS vs RR: Balanced accuracy=85%, Sensitivity=87%, Specificity=83% (SVM).<br>CIS vs RR+SP: Balanced accuracy=92%, Sensitivity=93%, Specificity=90% (SVM).<br>RR vs PP: Balanced accuracy=81% (SVM and LDA), Sensitivity=76%, Specificity=86% (SVM), Sensitivity=84%, Specificity=78% (LDA).<br>RR vs SP: Balanced accuracy=87%, Sensitivity=85%, Specificity=88% (SVM)	Leave-one-patient-out cross-validation
Kocevar et al. 2016 [23]	No	Disease Subtype	Supervised	Support Vector Machine	N=90 (n(MS)=64, n(HC)=26)	MRI Data	HC vs CIS: F-Measure=91·8%, Precision=92%, Recall=91·7%.<br>CIS vs RR: F-Measure=91·8%, Precision=92%, Recall=91·7%.<br>RR vs PP: F-Measure=75·6%, Precision=75·6%, Recall=75·6%.<br>RR vs SP: F-Measure=85·4%, Precision=85·5%, Recall=85·4%.<br>SP vs PP: F-Measure=66·7%, Precision=67·5, Recall=65·9.<br>CIS vs RR vs SP: F-Measure=70·6%, Precision=71·3%, Recall=70·0%	10-fold cross-validation

Paper	Multiple AIDs Studied	Prediction or Classification Task	ML Type	Machine Learning Method	Study Size (N)	Type of Data	Best Results (Metrics) Reported from validation or cross-validation, and where conducted, the test set.	Cross-Validation
Kosa et al. 2016 [24]	No	Disease Progression	Supervised	CombiWISE (algorithm combines disability scoring systems)	N=408	Clinical and MRI data	.	Hold-out validation
Baranzini et al. 2015 [25]	No	Disease progression	Supervised	Random Forest	N=155	RNA biomarkers, Clinical, MRI Data	Accuracy=0·68, Sensitivity=0·22, Specificity=0·88	Hold-out validation
Wottschel et al. 2015 [26]	No	Disease Progression	Supervised	Support Vector Machine	N=74	Clinical and MRI Data	1 year follow-up: Accuracy=71·4%, Sensitivity=77%, Specificity=66%.<br>3 year follow up: Accuracy=68% Sensitivity=60%, Specificity=76%	Leave-one-out cross-validation
Crimi et al 2014 [27]	No	Disease Progression	Supervised and Unsupervised	Spectral clustering and Least squares linear regression	N=25	MRI Data	R^2=0·9	Leave-one-patient out cross-validation
Sweeney et al. 2014 [28]	No	Image Segmentation	Supervised	Methods Analysed: Logistic Regression, Neural Network, Support Vector Machine, Quadratic Discriminant Analysis, Linear Discriminant Analysis, Gaussian Mixture Model, k Nearest Neighbour, Random Forest, Super Learner	N=98	MRI Data	.	Hold-out validation
Taschler et al. 2014 [29]	No	Disease Subtype	Supervised	Bayesian Spatial Generalized Linear Mixed Model or Log Guassian Cox Process	N=250	MRI Data	Bayesian Spatial Generalized Linear Mixed Model: Accuracy=0·895 (overall), 0·851 (average over all subtypes).<br>Log Guassian Cox Process: Accuracy=0·748 (overall), 0·823 (average over all subtypes)	Leave-one-out cross-validation
Alaqtash et al. 2011 [30]	No	Diagnosis and Disease Severity	Supervised	Nearest Neighbour Classifier (k Nearest Neighbours) or Artificial Neural Network	N=20 (n(HC)=12, n(spastic diplegic cerebral palsy)=4, n(RRMS)=4)	Clinical (Ground Reaction Forces; Gait Assessment) Data	Accuracy=95%, Sensitivity=96%, Specificity=95%	Leave-one-out cross-validation
Goldstein et al. 2010 [31]	No	Risk of Disease	Supervised	Random Forest	N=3362 (n(MS)=931, n(HC)=2431)	GWAS Data	.	Out-of-bag Error
Corvol et al. 2008 [32]	No	Risk of Disease	Supervised and Unsupervised	Hierarchical Clustering and Support Vector Machine	N=62 (n(CIS)=34, n(HC)=28)	Clinical, Microarray Data	Hierarchical Clustering of high-risk group: Sensitivity=92%, Specificity=86%.<br>Support vector machine on high-risk group: Accuracy=86%, Precision=78%, Negative Predictive Value=90%	10-fold cross-validation

Paper	Multiple AIDs Studied	Prediction or Classification Task	ML Type	Machine Learning Method	Study Size (N)	Type of Data	Best Results (Metrics) Reported from validation or cross-validation, and where conducted, the test set.	Cross-Validation
Briggs et al. 2010 [33]	No	Risk of Disease	Supervised	Random Forest	N=12566 (n(test)=1343 MS + 1379 HC, n(val)=2624 MS + 7220 HC)	SNP Data	.	Independent validation dataset
Commowick et al. 2018 [34]	No	Image Segmentation	Supervised	Consensus Model	N=53	MRI Data	Dice Score~0·63, F1-score~0·5	Hold-out validation
Ohanian et al. 2016 [35]	No	Disease Classification	Supervised	Decision Tree	N=460	Questionnaire Data	Accuracy=81·2% (MS & ME or CFS), 84·0% (ME or CFS), 79·2% (MS correctly categorised)	.
Salem et al. 2018 [36]	No	Diagnosis and Disease Monitoring	Supervised	Logistic Regression	N=60	MRI Data	Dice similarity coefficient=0·56 (segmentation), 0·77 (detection), F-score=0·806, Sensitivity=74·3%, Specificity=88·14%	Leave-one-out cross-validation
Cabezas et al. 2014 [37]	No	Disease Progression	Supervised	BOOST (ensemble classifier)	N=45 (three hospitals)	MRI Data	Median Dice Score=0·17 (hospital 1), 0·56 (hospital 2), 0·52 (hospital 3)	Leave-one-out cross-validation
Zhang et al. 2016 [38]	No	Diagnosis	Supervised	k Nearest Neighbours	N=38 and enrolled unspecified number of HCs age and gender matched	MRI Data	Accuracy=97·94%, Precision=99·09%, Sensitivity=96·15%, Specificity=99·32%	10-fold cross-validation
Birenbaum et al. 2017 [39]	No	Diagnosis and Disease Monitoring	Supervised	Convolution Neural Network	N=19 (training n=5, test n=14)	Clinical (MRI, longitudinal) Data	Cross-validation: Dice Score=0·727<br>Test Set: Dice Score=0·627	Leave-one-out cross-validation, independent test set
Morrison et al. 2016 [40].	No	Disease Monitoring	Supervised	Customized randomized forests and novel ensembles of randomized support vector machines	N=1041 videos	Movement Tests Data	Dice Score > 80%	.
Liu et al. 2015 [41]	No	Disease Progression	Unsupervised	Constraint-based clustering	N=266	Clinical Data	.	.
**Rheumatoid Arthritis**								
Chin et al. 2018 [42]	No	Risk of Disease	Supervised and Unsupervised	Non-negative Matrix Factorisation, Support Vector Machine	N=922,199 (n(RA)=1007, n(HC)=921,192)	Medical Diagnostic Database	Accuracy ~72%, Sensitivity~74%, Specificity~70%	10-fold cross-validation
Chocholova et al. 2018 [43]	No	Diagnosis and Disease Subtype	Supervised	Artificial Neural Network	N=100 (n(Seropositive RA)=31, n(Seronegative RA)=16, n(HC)=53	Immunoassay (Serum Samples) Data	Seropositive RA vs non-RA: AUC=0.96<br>Seronegative RA vs non-RA: AUC=0.86	Hold-out validation, testing set
Wu et al. 2018 [44]	No	Diagnosis	Supervised	Logistic Regression	N=806 (n(HC)=383, n(T2D)=170, n(RA)=130, n(Liver Cirrhosis)=123)	Microbiome and Clinical Data	AUC=0·96, F1-score=0·92	5-fold cross-validation

Paper	Multiple AIDs Studied	Prediction or Classification Task	ML Type	Machine Learning Method	Study Size (N)	Type of Data	Best Results (Metrics) Reported from validation or cross-validation, and where conducted, the test set.	Cross-Validation
Joo et al. 2017 [45]	No	Disease Progression	Supervised	Support Vector Machine	N=773 (n(train and validate)=374, n(test)=399)	GWAS & Clinical Data	Cross-validation: AUC=0·7822, Accuracy=0·7481, Sensitivity=0·7644, Specificity=0·7318. Independent Test Data: Accuracy=0·6143	10-fold cross-validation, Independent Test Datat
Andreu-Perez et al. 2017 [46]	No	Disease Monitoring	Supervised	Dichotomous Mapped Forest	N=30 (n(RA)=10, n(HC)=20)	Movement Data	Accuracy 95%, F-score 81%	Leave-one-subject-out cross-validation
Orange et al. 2018 [47]	No	Disease Subtype	Both	Consensus Clustering and Support Vector Machine	N=129 (n(RA)=123, n(OA)=6)	RNA sequence and Histology Data	AUC=0·88 (high inflammatory vs other), 0·71 (low inflammatory vs other), 0·59 (mixed subtype vs other)	Leave-one-out cross-validation
Ahmed et al. 2016 [48]	No	Diagnosis	Supervised	Random Forest	N=172 (n(early OA)=46, n(early RA)=45, n(non-RA)=42, n(advanced OA)=17, n(advanced RA)=22)	Plasma amino acid analyte Data	Disease vs HC. Training set Cross-validation: AUC=0·99 Sensitivity=0·92, Specificity=0·91. Test set Cross-validation: AUC=0·96, Sensitivity=0·89, Specificity=0·9.<br>Validation test set: AUC=0·77, Sensitivity=0·73, Specificity=0·72.<br><br>Early RA classification. Training set Cross-validation: AUC=0·91, Sensitivity=0·8, Specificity=0·78.<br>Test set Cross-validation: AUC=0·87, Sensitivity=0·77, Specificity=0·76.<br>Validation test set: AUC=0·62, Sensitivity=0·6, Specificity=0·61.	5-fold cross-validation on training set and test set.<br>Independent validation test set.
Miyoshi et al. 2016 [49]	No	Response to treatment	Supervised	Multilayer Perceptron	N=180	Clinical Data	AUC=0·75, Accuracy=92%, Sensitivity=96·7%, Specificity=75%	Hold-out validation
Yeo et al. 2016 [50]	No	Early Diagnosis	Supervised	Multivariate Analysis	N=48 (n(Uninflamed Controls)=10, n(Resolving Arthritis)=9, n(early RA)=17, n(established RA)=12)	Synovial mRNA Data	Established RA vs Uninflamed: AUC=0·996 Early RA vs Resolving RA: AUC=0·764	.
Zhou et al. 2016 [51]	No	Identification of Patients	Supervised	Random Forest and C5.0 Decision Tree	N=480788	EHR Data	Test dataset 1: Accuracy=92·29% Sensitivity=86·2%, Specificity=94·6%<br>Test dataset 2: Best-case scenario: Sensitivity=94%, Specificity=99·9%. Worst-case scenario: Sensitivity=83%, Specificity=99%	Two independent testing datasets
Lin et al. 2015 [52]	No	Identification of Patients	Supervised	Natural Language Processing and Classification Rules	N=600 (n(RA with liver toxicity)=170, n(RA)=430)	EMR Data	Cross-validation: F1-score=0·847, Precision=0·8, Recall=0·899<br>Test Set: F1-score=0·829, Precision=0·756, Recall=0·919	10-fold cross validation, independent test set

Paper	Multiple AIDs Studied	Prediction or Classification Task	ML Type	Machine Learning Method	Study Size (N)	Type of Data	Best Results (Metrics) Reported from validation or cross-validation, and where conducted, the test set.	Cross-Validation
Chen et al. 2013 [53]	No	Identification of Patients	Supervised	Active Learning and Support Vector Machine	N=376 (n(RA)=185, n(Controls)=191)	EHR Data	AUC > 0·95	5-fold cross-validation
Lin et al. 2013 [54]	No	Disease Severity	Supervised	Natural Language Processing and Support Vector Machine	N=2017 (n(train)=852, n(test set 1)=821, n(test set 2)=344)	EMR Data	Test set 1 AUC=0·831, F1-score=0·789. Test set 2 AUC=0·785, F1 score=0·761	10-fold cross validation on two test sets
Negi et al. 2013 [55]	No	Risk of Disease	Supervised	Support Vector Machine	N=3542 (n(train)=706 RA + 761 Controls, n(test)=927 RA + 1148 Controls)	SNP Data	AUC=0·93, Accuracy=88·7%	Cross validation used
Pratt et al. 2012 [56]	No	Early Diagnosis	Supervised	Support Vector Machine	N=173 (n(RA)= 47, n(non-RA)=64, n(undifferentiated arthritis)=62)	CD4 T Cell Transcriptome Data	Sensitivity=0·68, Specificity=0·7. Removing ACPA-positive subset: Sensitivity=0·85, Specificity=0·75	Hold out validation
Singh et al. 2012 [57]	No	Diagnosis	Supervised	Fuzzy Inference System	N=150	Clinical Data	.	.
Kruppa et al. 2012 [58]	No	Risk of Disease	Supervised	Random Forest in regression mode (Random Jungle)	N=1445 (n(RA)=707 and n(HC)=738)	GWAS Data	AUC=0·8925	Hold-out validation
Liu et al. 2011 [59]	No	Risk of Disease	Supervised	Random Forest	N=4880 (n(cohort 1)=908 RA + 1260 controls, n(cohort 2)= 952 RA + 1760 controls)	SNP Data	Accuracy=70%, Sensitivity=74%, Specificity=66%	Out of bag error, Independent validation cohort
Nair et al. 2010 [60]	No	Response to treatment	Supervised	Least Squares Kernel-Conjugate gradient algorithm	N=25 (n(RA)=8, n(OA)=10, n(HC)=7)	Electro-myographic Gait Data	Accuracy=91·07%, Sensitivity=81%, Specificity=82%	8-fold cross-validation
Briggs et al. 2010 [61]	No	Risk of Disease	Supervised	Random Forest and Logistic Regression	N= 4130	SNP Data	.	Hold-out validation
Niu et al. 2010 [62]	No	Diagnosis	Supervised	Boosted Decision Tree	N=143 (n(RA)=43, n(AID Controls)=50, n(HC)=50)	Mass Spectrometry (from serum)	Accuracy=85·7% (RA), 87·5% (autoimmune controls), 88·0% (HC). Sensitivity=85·71%, Specificity=87·76% (RA vs controls)	Hold-out validation
Geurts et al. 2005 [63]	Yes	Diagnosis	Supervised	Decision Trees (RA Boosting, IBD Extra-Trees)	N(RA)=206 (68 RA, 138 controls), N(IBD)=480 (240 IBD, 240 controls)	Mass Spectrometry (from serum)	RA: Sensitivity=83·82%, Specificity=94·93% IBD: Sensitivity=88·33%, Specificity= 91·63%	Leave-one-out cross-validation
de Seny et al. 2005 [64]	Yes	Early Diagnosis	Supervised	Decision Tree Boosting	N=103 (n(RA)=34, n(inflammatory controls)=20 PsA + 9 Asthma + 10 CrD, n(controls)=14 OA + 16 HC)	Mass Spectrometry (from serum)	RA vs controls: Sensitivity=85%, Specificity=91% (2 independent spectra), Sensitivity=94%, Specificity=90% (2 combined spectra). RA vs PsA: Sensitivity=94%, Specificity=86% (2 independent spectra), Sensitivity=97%, Specificity=76% (2 combined spectra).	Leave-one-out cross validation
Scheel et al. 2003 [65]	No	Early Diagnosis	Supervised	Neural Network, Method in [66]	N=22 patients, N=72 joints examined	Laser Imaging Data	Accuracy=86%, Sensitivity=80%, Specificity=89%	.

Paper	Multiple AIDs Studied	Prediction or Classification Task	ML Type	Machine Learning Method	Study Size (N)	Type of Data	Best Results (Metrics) Reported from validation or cross-validation, and where conducted, the test set.	Cross-Validation
Gronsbell et al. 2018 [67]	No	Identification of Patients	Supervised and Unsupervised	Unsupervised (clustering based) Feature Selection and Sparse Regression	N=435	EMR Data	AUC=0·928	Independent validation dataset
Gossec et al. 2018 [68]	Yes	Disease Monitoring	Supervised	Multiclass Selective Naïve Bayes Classifier	N=155 (82 RA, 73 axSpA)	Physical Activity Data	Sensitivity=95·7%, Specificity=96·7%	Hold-out validation
Lezcano-Valverde et al. 2017 [69]	No	Mortality	Supervised	Random Survival Forests	N=1741	Demographic & Clinical Data	1 year follow-up: Sensitivity=0·79, Specificity=0·8.<br>7 year follow up: Sensitivity=0·43, Specificity=0·48.	Hold-out validation
Gonzalez-Recio et al. 2009 [70]	No	Risk of Disease	Supervised	Information gain/entropy reduction criteria and Bayesian threshold LASSO	N=2062 (n(cases)=868, n(controls)=1194)	SNPs	.	5-fold cross-validation
Heard et al. 2014 [71]	No	Early Diagnosis	Supervised	Artificial Neural Network and Decision Tree	ANN: N=300 (n(HC)=98 n(OA)=101, n(RA)=101)<br>DT: N=298 (n(HC)= 100, n(OA)=100, n(RA)=98)	Clinical (Inflammatory cytokine expression, serum samples) Data	ANN: Sensitivity=100% (HC), 100% (OA), 100% (RA), Specificity=100% (HC), 100% (OA), 100% (RA) for all cytokines and significant cytokines.<br>DT: Sensitivity=100% (HC), 100% (OA), 95% (RA), Specificity=96% (HC), 97% (OA), 100% (RA) for all cytokines.	Hold-out validation, independent testing set
Gronsbell et al. 2018 [72]	Yes	Identification of Patients	Semi-Supervised	Semi-supervised approach	N(RA)=44014 (500 labelled, 43514 unlabelled), N(MS)=12198 (455 labelled, 11743 unlabelled)	EMR Data	AUC=94·93 (RA), 93·94 (MS)	10-fold cross-validation
Van Looy et al. 2006 [73]	No	Response to treatment	Supervised	Multilayer Perceptron or Support Vector Machine	N=511	Clinical Data	All Cases: AUC=0·772, Sensitivity=0·95, Specificity=0.402 or Sensitivity=0·265, Specificity=0·95 (MLP).<br>Complete Cases, MLP: AUC=0·854, Sensitivity=0.95, Specificity=0·548 or Sensitivity=0·462, Specificity=0·95.<br>Complete Cases, SVM: AUC=0·863, Sensitivity=0·95, Specificity=0·507 or Sensitivity=0·308, Specificity=0·95.<br>Expectation Maximisation, MLP: AUC=0·813, Sensitivity=0·95, Specificity=0·411, or Sensitivity=0·412, Specificity=0·95.<br>Expectation Maximisation, SVM: AUC=0·804, Sensitivity=0·95, Specificity=0·402, or Sensitivity=0·412, Specificity=0·95.	.
Wyns et al. 2004 [74]	No	Early Diagnosis	Supervised and Unsupervised	Kohonen Neural Network (includes Self Organising Maps)	N=160 (n(RA)=51 RA, n(SpA)=43, n(other)=26, n=40 with no definite diagnosis)	Clinical Data	Accuracy=62·3%, 65·3% (without undetermined samples)	Hold-out validation
**Inflammatory Bowel Disease**								
Waljee et al. 2018 [75]	No	Disease Progression	Supervised	Random Forest	N =20368	Clinical Data	Predict Hospitalisation and Corticosteroid Prescriptions. IBD: AUC=0·87, Sensitivity=74-	Hold-out validation

Paper	Multiple AIDs Studied	Prediction or Classification Task	ML Type	Machine Learning Method	Study Size (N)	Type of Data	Best Results (Metrics) Reported from validation or cross-validation, and where conducted, the test set.	Cross-Validation
							80%, Specificity=80-82%. UC: AUC=0·84. CrD=0·85. IC=0·82.<br>Predict Corticosteroid Prescription Only, IBD: AUC=0·9<br>Predict Hospitalisation and Corticosteroid Prescriptions (12 month outcome): AUC=0·9	
Mossotto et al. 2017 [76]	No	Disease Subtype	Supervised and Unsupervised	Support Vector Machine, Hierarchical Clustering	N=287 Training and testing: N=210 (n(CrD)=178, n(UC)=80, n(IBDU)=29 (only reclassified))	Clinical Data	Cross-validation: AUC=0·87, Accuracy=82·7%, Precision=0·91, Recall=0·83, F1-score=0·87.<br>Independent test set: Accuracy=83·3%, Precision=0·86, Recall=0·83, F1-score=0·84	5-fold cross validation, independent test set
Maeda et al. 2018 [77]	No	Disease Severity	Supervised	Support Vector Machine	N=187	Endocytoscopic Image Data	Accuracy=91%, Kappa=1, Sensitivity=74%, Specificity=97%	Hold-out validation
Douglas et al. 2018 [78]	No	Diagnosis and Response to Treatment	Supervised	Random Forest	N=771 (n (test)=40 (n(CrD)=20, n(HC)=20). n(validation, diagnosis only) = 731 (444 CrD, 287 control))	Metagenomic Data	Diagnosis: Accuracy=84·2%.<br>Independent Validation (diagnosis): Accuracy 73·2%.<br>Treatment Response: Accuracy 77·8%.	Out of bag error, Leave-one-out cross-validation, Independent test data (diagnosis only)
Jain et al. 2017 [79]	No	Disease Progression	Supervised	Decision Tree	N=179	Clinical Data	Colectomy Prediction: Accuracy=77%, Sensitivity=75%, Specificity=80%.<br>Steroid Dependence: Accuracy=75%, Sensitivity=69%, Specificity=80%.	Hold-out Validation
Waljee et al. 2017 [80]	No	Response to Treatment	Supervised	Random Forest	N=1080	Clinical Data	Objective Remission: AUC=0·79, Sensitivity=70·6%, Specificity=73·8%.<br>Non-adherence: AUC=0·84, Sensitivity=70·6%, Specificity=85·0%.<br>Shunting: AUC=0·78, Sensitivity=65·2%, Specificity=79·0%.	Out of bag error, Hold-out validation
Isakov et al. 2017 [81]	No	Risk of Disease	Supervised	Combined Model (elastic net regularised generalised linear model, extreme gradient boosting, support vector machine, random forest)	N = 513 (n(CrD)=180, n(UC)=149, n(colorectal neoplasms)=94, n(normal tissue)=90)	Gene Expression Data	AUC=0·829, Accuracy=0·808, Sensitivity=0·577, Specificity=0·880	5-fold cross-validation
Kang et al. 2017 [82]	No	Response to Treatment	Supervised	Gene Regulatory Network-based Regularized Artificial Neural Network (GRRANN)	N=46	Gene Expression Data	Balanced Accuracy≈0·8	5-fold cross validation, Hold-out validation
Waljee et al. 2018 [83]	No	Response to Treatment	Supervised	Random Forest	N=491	Clinical Data	AUC=0·73, Sensitivity=0·72, Specificity=0·68	Hold-out validation

Paper	Multiple AIDs Studied	Prediction or Classification Task	ML Type	Machine Learning Method	Study Size (N)	Type of Data	Best Results (Metrics) Reported from validation or cross-validation, and where conducted, the test set.	Cross-Validation
Pal et al. 2017 [84]	No	Risk of Disease	Supervised	Consensus Method (Naïve Bayes, Logistic Regression, Random Forest)	N=111 (n(CrD)=64, n(HC)=47)	GWAS Data, Exome Data to impute genotypes.	AUC=0·72	Hold-out validation
Eck et al. 2017 [85]	No	Diagnosis	Supervised	Support Vector Machine or Random Forest	N=112 (n(IBD)=56, n(HC)=56)	Microbiota Data	Accuracy=81%	10-fold cross validation
Menti et al. 2016 [86]	No	Disease Progression	Supervised	Bayesian Networks	N=152	Clinical Data and Selected Genetic Data	AUC=0·95, Accuracy=0·89, Sensitivity=0·78, Specificity=0·94	10-fold cross validation
Hubenthal et al. 2015 [87]	No	Diagnosis	Supervised	Support Vector Machine	N=273 (n(CrD)=37, n(UC)=32, n(HC)=92, n(COPD)=23, n(MS)=23, n(pancreatitis)=35, n(sarcoidosis)=32)	MicroRNA Expression Data	AUC=0·95, Balanced Accuracy=0·95, Sensitivity=1, Specificity=0·9	5-fold cross-validation
Niehaus et al. 2015 [88]	No	Disease Severity	Supervised and Unsupervised	Support Vector Machine, Hierarchical Clustering	N=501	Health Records, EMR Databases	Accuracy=68·7%, Sensitivity=59·1%, Specificity=78·4%	5-fold cross validation, testing dataset
Wei et al. 2013 [89]	No	Risk of Disease	Supervised	Logistic Regression	N=53,279 (n(CrD)=17,379, n(UC)=13,458, n(HC)= 22,442	GWAS Data	Cross Validation: AUC=0·864 (CrD) 0·83 (UC). Independent Test Set: AUC=0·864 (CrD), 0·826 (UC)	10-fold cross validation, independent testing dataset
Cui et al. 2013 [90]	No	Diagnosis	Supervised	Support Vector Machine	N=124 (n(IBD)=25, n(HC)=99)	Metagenomic Data	Accuracy=88%, Sensitivity=92%, Specificity=84%	Leave-one-out cross-validation
Waljee et al. 2010 [91]	No	Response to Treatment	Supervised	Random Forest	N=346	Clinical Data	AUC=0·856 (response), 0·813 (non-adherence), 0·797 (shunting)	10-fold cross validation, validation data set
Firouzi et al 2007 [92]	No	Disease Progression	Supervised	Decision Tree	N=160 (121 UC, 39 CrD)	Clinical Data	Accuracy=88·2% (UC), 89·8% (CrD), 86·5% (IBD), Sensitivity=67·6% (UC), 82·8% (CrD), 65·7% (IBD), Specificity=96·3% (UC), 95·2% (CrD), 96·3% (IBD), Matthew's Correlation Coefficients=0·69 (UC), 0·79 (CrD), 0·68 (IBD)	10-fold cross-validation
Ozawa et al. 2018 [93]	No	Disease Severity	Supervised	Neural Network	N= 30,285 images, N=558 patients	Colonoscopy White-light Image Data	Mayo 0 vs Mayo 1-3: AUC=0·86. Mayo 0-1 vs Mayo 2-3: AUC=0·98	Hold-out validation
Reddy et al. 2018 [94]	No	Disease Severity	Supervised	Gradient Boosting Machines	N=82	EHR Data	AUC=92·82%	10-fold cross-validation
Forbes et al. 2018 [95]	Yes	Diagnosis	Supervised	Random Forest	N=102 (n(CrD)=20, n(UC)=19, n(MS)=19, n(RA)=21, n(HC)=23)	Microbiota Data	Diseased vs HC: AUC=0·93, Balanced Accuracy=0·84. Breakdown per inflammatory disease found in paper	Out of bag error

Paper	Multiple AIDs Studied	Prediction or Classification Task	ML Type	Machine Learning Method	Study Size (N)	Type of Data	Best Results (Metrics) Reported from validation or cross-validation, and where conducted, the test set.	Cross-Validation
Doherty et al. 2018 [96]	No	Response to treatment	Supervised	Random Forest	N=306 (n(CrD treated)=232, n(CrD untreated)=74)	Microbial Genome Data and Clinical Data	Remission: AUC=0·844, Sensitivity=0·774, Specificity=0·831.<br>Response: AUC=0·733 Sensitivity=0·684, Specificity=0·724	.
Han et al. 2018 [97]	No	Disease subtype	Supervised	Random Forest	N=163 (n(train)=24 CrD, 59 UC, n(Validation set 1)=5 CrD , 7 UC, n(Validation set 2)=14 CrD, 10 UC, n(Validation set 3)=11 CrD, 5 UC, n(Validation set 4)=13 CrD, 15 UC) Biopsy Samples	Gene Expression Data	Validation set 1: AUC=0·829<br>Validation set 2: AUC=0·764<br>Validation set 3: AUC=0·836<br>Validation set 4: AUC=0·849	Hold-out validation
Daneshjou et al. 2017 [98]	No	Risk of Disease	Supervised	Metaclassifier	N=111 (n(CrD)=64, n(HC)=47)	Exome-sequencing data	AUC=0·78	Cross-validation performed
Giollo et al. 2017 [99]	No	Risk of Disease	Supervised	Support Vector Machine or Ensemble Classifier	N=111 (n(cases)=64, n(controls)=47)	Genetic Data	AUC=0·6 (SVM), 0·66 (Ensemble Classifier)	Cross validation performed
Yu et al. 2017 [100]	Yes	Identification of Patients	Supervised	Natural Language Processing	N= 2393 (435 RA, 758 CAD, 600 UC, 600 CrD)	Electronic Medical Records Data	AUC~0·94 (RA), ~0·95 (CrD), ~0·95 (UC) F-score ~0·71 (RA), ~0·83 (CrD), ~0·89 (UC)	.
Wisittipanit et al. 2015 [101]	No	Diagnosis	Supervised	Support Vector Machine	N=425 (n(CrD)=101, n(UC)=89, n(HC)=235 HC)	LH-PCR (Microbiome) Data	AUC=0·73 (CrD), 0·78 (UC), 0·77 (HC), Accuracy=78·18% (CrD), 79·71% (UC), 75·62% (HC)	5-fold cross validation
Ahmed et al. 2017 [102]	No	Diagnosis	Supervised	Neuro-Fuzzy Automated Classifier	N=387 (n(CrD)=144, n(HC)=243)	Genetic Data	Accuracy=97·67%, Sensitivity=96·07%, Specificity=100%	Hold-out validation, testing set
Mahapatra et al. 2016 [103]	No	Image Segmentation	Semi-Supervised	Random Forest-based Classifier	N=70 (CrD)	MRI Data	Dice metric=92·4%, Hausdorff=7mm	5-fold cross validation
Mahapatra et al. 2016 [104]	No	Image Segmentation	Supervised	Random Forest	N=50 (CrD)	MRI Data	Dice metric=91·7%, Hausdorff=7.4mm	5-fold cross validation
**Type 1 Diabetes**								
Stawiski et al. 2018 [105]	No	Diagnosis	Supervised	Artificial Neural Network	N=315	Clinical Data	R^2=0·6455	Hold-out validation
Ben Ali et al. 2018 [106]	No	Disease Management	Supervised	Artificial Neural Network	N=12 patients, N=1344 samples	CGM Data	Average RMSE=6·43 (mg/dL)	Hold-out validation
Perez-Gandia et al. 2018 [107]	No	Disease Management	Supervised	Decision Support System with Artificial Neural Network	N= 21 patients, longitudinal analysis	Clinical Data	.	Hold-out validation

Paper	Multiple AIDs Studied	Prediction or Classification Task	ML Type	Machine Learning Method	Study Size (N)	Type of Data	Best Results (Metrics) Reported from validation or cross-validation, and where conducted, the test set.	Cross-Validation
Maulucci et al. 2017 [108]	No	Diagnosis and Disease Monitoring	Supervised	Decision Support System	N=26	RBC Image Data	Control): Accuracy=1, Precision=1, Recall=1, F1-score=1.<br>T1D: Accuracy=1, Precision=1, Recall=1, F1-score=1.<br>T1D with complications: Accuracy=1, Precision=1, Recall=1, F1-score=1.	Leave-one-person-out cross-validation
Siegel et al. 2017 [109]	No	Disease Management	Supervised	Linear Discriminant Analysis	N=52 patients, N=128 samples.	VOCs	AUC=0·895, Sensitivity=91%, Specificity=84%	Leave-one-out cross-validation
Zhao et al. 2016 [110]	No	Risk of Disease	Supervised	LASSO (regression)/OOR (developed method)	N=1418 (n(T1D)=962 T1D, n(controls)= 448	Genetic Data	AUC=0·89	Hold-out validation
Georga et al. 2015 [111]	No	Disease Management	Supervised	KOS-ELM (online sequential extreme learning machine kernels)	N=15, longitudinal analysis	Clinical Data	Case 1: RMSE=16·6 (mg/dl)<br>Case 2: RMSE=10·9 (mg/dl)<br>Case 3: RMSE=8·5 (mg/dl)	10-fold cross validation
Georga et al. 2013 [112]	No	Disease Management	Supervised	Support Vector Regression	N=15 patients, longitudinal analysis	Clinical Data	Nocturnal: Sensitivity=0·94, Precision=0·98 (30 minutes and 60 minutes).<br>Diurnal: Sensitivity=0·92, Precision=0·93 (30 minutes), Sensitivity=0·96, Precision=0·97 (60 minutes)	10-fold cross validation
Marling et al. 2013 [113]	No	Disease Management	Supervised	Support Vector Machine Regression	N=19 patients, N=262 CGM plots	CGM Data	Accuracy=90·1%, Sensitivity=97%, Specificity=74·1%	10-fold cross-validation
Nguyen et al. 2013 [114]	No	Risk of Disease	Supervised	RIPPER (decision rules) and Logistic Regression Method (Predict DQ types without DR type information)	N=10579 (n(train)=7405, n(test)=3174)	SNP Data	Independent Test Dataset. Predict HLA Types: AUC=0·997 Accuracy=99·3%.<br>Predict High Risk HLA types AUC=0·995 Accuracy=99·8%.<br>Predict high risk subtype (DRB1*03:01-DQA1*05:01-DQB1*02:01): AUC=0·998, Accuracy=99·8%.<br>Predict DQ Types without DR type information: AUC=0·98.	10-fold cross validation, independent test dataset
Wei et al. 2009 [115]	No	Risk of Disease	Supervised	Support Vector Machine	N=8438 (n(WTCCC-T1D)=1963 cases + 1480 controls ,n(CHOP/Montreal-T1D)= 1008 cases + 1000 controls, n(GoKinD-T1D)=1529 cases + 1458 controls)	GWAS Data	WTCCC-T1D dataset: AUC=0·89, Sensitivity=0·87, Specificity=0·75. CHOP/Montreal-T1D dataset: AUC=0·83, GoKinD-T1D dataset: AUC=0·84	5-fold cross-validation
Jensen et al. 2014 [116]	No	Disease Management	Unsupervised	Pattern Classification Algorithm	N=10 patients, longitudinal measurements (20 x sessions with Professional CGM)	CGM Data	Sensitivity=78%, Specificity=96%, (All hypoglycaemic events detected, 1 false positive)	.

Paper	Multiple AIDs Studied	Prediction or Classification Task	ML Type	Machine Learning Method	Study Size (N)	Type of Data	Best Results (Metrics) Reported from validation or cross-validation, and where conducted, the test set.	Cross-Validation
Schwartz et al. 2008 [117]	No	Disease Management	Supervised	Case-based reasoning	N=12 patients, longitudinal measurements	Clinical Data	.	.
Cordelli et al. 2018 [118]	No	Diagnosis and Diseases Monitoring	Supervised	Support Vector Machine	N=27 (n(HC)=8, n(T1D)=10, n(T1D with complications)=9	RBC Images	F1 score=1, Precision=1, Recall=1 (for HC, T1D, and T1D with complications)	Leave-one-person-out cross-validation
Sampath et al. 2016 [119]	No	Disease Management	Supervised	Aggregating ranking algorithms in machine learning	N=213 (n(DIAdvisor)=34, n(ChildrenData)=179)	Clinical Data	Sensitivity=77·03%, Specificity=83·46%	Independent validation dataset
Georga et al. 2015 [120]	No	Disease Management	Supervised	Random Forest (feature selection), Support Vector Regression or Gaussian processes	N=15 patients, longitudinal measurements	Clinical Data	30min prediction horizon: SVR RMSE=5·7, GP RMSE=5·6; 60min prediction horizon: SVR RMSE=6·4, GP RMSE=6·3	10-fold cross-validation
Ling et al. 2016 [121]	No	Disease Management	Supervised	Extreme learning machine-based neural network	N=16 patients, N=589 samples	Clinical Data	Gamma value=70·8%, Sensitivity=78%, Specificity=60%	Noted by researchers that cross-validation is not required.
**Systemic Lupus Erythematosus**								
Ceccarelli et al. 2018 [122]	No	Disease Progression	Supervised	Logistic Regression	N=120	Clinical Data	AUC=0·806	Leave-one-out cross-validation
Turner et al. 2017 [123]	No	Identification of Patients	Supervised	Natural Language Processing and Neural Network or Random Forest	N=662 (n(SLE)=332, n(HC)=340)	EHR Data	AUC=0·974 (Neural Network), 0·988 (RF), Accuracy=92·1% (Neural Network), 95% (Random Forest)	5-fold cross-validation
Ceccarelli et al. 2017 [124]	No	Disease Progression	Supervised	Recurrent Neural Networks	N=132 (n(develop chronic damage)=38, n(no chronic damage)=94)	Clinical Data	AUC=0·77, Sensitivity=0·74, Specificity=0·76	8-fold cross-validation
Kan et al. 2016 [125]	No	Disease Progression	Unsupervised	Cluster Analysis	N=1611	Demographic & Drug Treatment	.	Cross-validation not recommended for cluster analysis
Wolf et al. 2016 [126]	No	Treatment Response	Supervised	Random Forest	N=140 (n(non-responders)=103, n(responders)=37)	Urine Biomarkers	AUC=0·79, Sensitivity=0·76, Specificity=0·73	Cross-validation not required for Random Forest
Guy et al. 2012 [127]	No	Risk of Disease	Supervised	Bagged Alternating Decision Trees	N=6728 (1846 SLE + 1825 Controls)	SNPs	.	.
Tang et al. 2011 [128]	No	Mortality	Supervised	Logistic Regression	N= 3313	Clinical Record Data	AUC=0·74	10-fold cross-validation
Armananzas et al. 2009 [129]	Yes	Diagnosis	Supervised and Unsupervised	Consensus Method	N=14 (n(HC)=6, n(SLE)=3, n(PAPS)=5)	Microarray Expression Data	.	10-fold cross-validation
Huang et al. 2009 [130]	No	Diagnosis	Supervised	Decision Tree	N=232 (n(SLE)=64, n(AID controls)=85, n(HC)=83)	Serum Proteome Data	SLE: Accuracy=78·1%, Sensitivity=78·1%, Specificity=96·3%<br>AID Controls: Accuracy=85·8%, Sensitivity=85·7%, Specificity=86·7%	Hold-out validation

Paper	Multiple AIDs Studied	Prediction or Classification Task	ML Type	Machine Learning Method	Study Size (N)	Type of Data	Best Results (Metrics) Reported from validation or cross-validation, and where conducted, the test set.	Cross-Validation
							HC: Accuracy=90%, Sensitivity=90%, Specificity=96%.	
Murray et al. 2018 [131]	No	Identification of Patients	Supervised	Logistic Regression	N=17057 (n(SLE)=583, n(control)=16174, n(potential SLE)=150, n(random)=150)	EHR Data	AUC=0·97, Accuracy=0·92, Precision=0·85, Recall=0·97	Hold-out validation
Reddy et al. 2018 [132]	No	Disease Progression	Supervised	Recurrent Neural Network	N=9457	EHR Data	AUC=0·7, Accuracy=70·54%, Sensitivity=74·49%, Specificity=56·61%	Hold-out validation
Tang et al. 2018 [133]	No	Disease Progression	Supervised	Random Forest and Multilinear Regression	N=173	Clinical Data	Random Forest, multi-classifier: Accuracy=53·7% (Class II), 56·2% (Class III&IV):56·2%, 40·1% (Class V).<br>Random Forest, binary classifier: Accuracy=56·2% (Class II), 63·7% (Class III&IV), 61% (Class V). Multilinear regression: CI prediction: Q^2=0·746, R^2=0·771. AI prediction: Q^2=0·516, R^2=0·576.	5-fold cross validation (Predicting AI and CI)
Scully et al. 2010 [134]	No	Diagnosis	Supervised	Naïve Bayesian Classifier and Support Vector Machine	N=27	MRI Data	Leave-one-out training data: Sensitivity=94·3%, Specificity= 93·1%<br>Test data: Sensitivity=94·3%, Specificity=93.9%	Leave one out cross validation, Test dataset
Davis et al. 2013 [135]	No	Risk of Disease	Supervised	Random Jungle, ReliefF or evaporative cooling	N=404 (n(SLE)=209, n(HC)=195)	Exome Data	.	.
**Psoriasis and Psoriatic Arthritis**								
Wang et al. 2016 [136]	No	Diagnosis	Supervised	Random Bits Forest (Neural Network, Boosting, Random Forest)	N=2723 (n(train)=915 cases + 675 controls; n(test)=431 cases + 702 controls)	GWAS Data	Cross-validation: AUC=0·6739, Accuracy=0·639, Sensitivity=0·6317, Specificity=0·649.<br>Test Dataset: AUC=0·7239, Accuracy=0·692, Sensitivity=0·6543, Specificity=0·7151.	10-fold cross validation, independent testing dataset
George et al. 2018 [137]	No	Disease Severity	Supervised and Unsupervised	Unsupervised Feature Learning, Random Forest	N=676 images, N=44 patients	Digital Image Data	F1-score=0·71	10-fold cross validation
Shrivastava et al. 2017 [138]	No	Disease Severity	Supervised	Support Vector Machine	N=670 images, N=110 patients	Digital Image Data	AUC=0·998, Accuracy=99·84%, Sensitivity=99·76%, Specificity=99·99%	10-fold cross validation
Shrivastava et al. 2016 [139]	No	Diagnosis	Supervised	Support Vector Machine	N=540 (n(HC)=270, n(P)=270) images, N=30 patients.	Digital Image Data	AUC=1, Accuracy=100%, Sensitivity=100%, Specificity=100%	10-fold cross validation
Shrivastava et al. 2016 [140]	No	Disease Severity	Supervised	Support Vector Machine	N=848 images, N=65 patients	Digital Image Data	Accuracy=99·92%	10-fold cross validation
Shrivastava et al. 2015 [141]	No	Diagnosis	Supervised	Support Vector Machine	N=540 (n(HC)=270, n(P)=270) images, N=30 patients.	Digital Image Data	AUC=0·999, Accuracy=99·94%, Sensitivity=99·93, Specificity=99·96%	10-fold cross validation

Paper	Multiple AIDs Studied	Prediction or Classification Task	ML Type	Machine Learning Method	Study Size (N)	Type of Data	Best Results (Metrics) Reported from validation or cross-validation, and where conducted, the test set.	Cross-Validation
Cowen et al. 2007 [142]	No	Diagnosis	Supervised	Partial Least Squares Regression, Support Vector Machine and C5.0 Decision Tree	N=148 (n(tumour-stage MF)=45, n(psoriasis)=56, n(HC)=47)	Proteomic Data from Serum	Tumour-Stage MF vs Psoriasis: Sensitivity=78·57%, Specificity=93·75% (Ciphergen), Sensitivity=78·57, Specificity=86·67% (PrOTOF). Psoriasis vs HC: Sensitivity=93·75%, Specificity=75% (Ciphergen), Sensitivity=86·67%, Specificity=76·92%. (PrOTOF).	10-fold cross validation, independent testing dataset
Raina et al. 2016 [143]	No	Disease Severity	Supervised	Linear Discriminant Analysis	N=20 patients, N=80 images	Digital Image Data	Accuracy=48·75%, Kappa=0·4203	Leave-one-out cross-validation
Shrivastava et al. 2015 [144]	No	Diagnosis	Supervised	Support Vector Machine	N=540 (n(HC)=270, n(P)=270) images, N=30 patients.	Digital Image Data	AUC=1, Accuracy=99·81%, Sensitivity=99·26%, Specificity=97·04%	Jack Knife (N fold) cross-validation
Shrivastava et al. 2016 [145]	No	Diagnosis	Supervised	Support Vector Machine	N=540 (n(HC)=270, n(P)=270) images, N=30 patients.	Digital Image Data	AUC=0·99, Accuracy=99·39%, Sensitivity=99·43%, Specificity=99·35%	10-fold cross-validation
Patrick et al. 2018 [146]	Yes	Risk of Disease and Disease Progression	Supervised	Conditional Inference Forest or Shrinkage Discriminant Analysis	N=22181 (n(PsV)=7855, n(PsA)=2703, n(PsC)=2681, n(HC)=8942)	GWAS Data	AUC=0·82 (cross validation and holdout test set)	Cross-validation performed, test set
**Coeliac Disease**								
Hujoel et al. 2018 [147]	No	Diagnosis	Supervised	Random Forest or Bagged Classification Trees	N = 408	EMR Data	AUC≈0·55	10-fold cross-validation
Arasaradnam et al. 2014 [148]	No	Diagnosis	Supervised	Logistic Regression	N=47 (n(D-IBS)=20, n(CeD)=27)	VOCs Data	AUC=0·91, Sensitivity=85%, Specificity=85%	Leave-one-out cross-validation
Tenorio et al. 2011 [149]	No	Diagnosis	Supervised	Bayesian Classifier (Average One-Dependence Estimator)	N=216 (CeD 46% of records in training data, 37% in test data)	Clinical Data	AUC=0·84, Accuracy=80%, Sensitivity=0·78, Specificity=0·80	10-fold cross-validation
Choung et al. 2018 [150]	No	Diagnosis and Disease Monitoring	Supervised	Random Forest (peptide selection), Support Vector Machine	Diagnosis: N= 468 (n(CeD)= 172, n(HC)=296). Monitoring: N= 465 (n(CeD treated, healed)=85, n(CeD treated, unhealed)=81, n(CeD, untreated)=82, n(HC)=217, n(disease controls)=27).	Peptide Data	Diagnosis: Accuracy=99%, Sensitivity=99%, Specificity=100%. Monitoring: Accuracy=90%, Sensitivity=84%, Specificity=95%	Hold-out validation (diagnosis only)
Chen et al. 2016 [151]	No	Diagnosis	Supervised	Logistic Model	N=1498 (n(CeD)=363, n(FP)=1135)	EHR Data	AUC=0·94, F1-score=0·92, Kappa=0·78, Precision=0·93, Recall=0·92	10-fold cross-validation
Ludvigsson et al. 2013 [152]	No	Diagnosis	Supervised	Natural Language Processing	N=496 (n(train)=327, n(test)=169)	EMR Data	F-measure 84·5%, Sensitivity=72·9%, Specificity=89·9%	Hold-out validation

Paper	Multiple AIDs Studied	Prediction or Classification Task	ML Type	Machine Learning Method	Study Size (N)	Type of Data	Best Results (Metrics) Reported from validation or cross-validation, and where conducted, the test set.	Cross-Validation
Amirkhani et al. 2018 [153]	No	Disease Severity	Supervised	Combined fuzzy cognitive map and possibilistic fuzzy c-means clustering algorithm	N=89	Clinical Data	Accuracy=91% (A), 90% (B1), 88% (B2)	Leave-one-out cross-validation
**Thyroid Disease**								
Ahmad et al. 2018 [154]	No	Diagnosis	Supervised	Hybrid model (linear discriminant analysis, k-nearest neighbour weighed preprocessing, adaptive neurofuzzy inference system)	N=3163 (n(hypo)=152, n(negative)=3011)	Clinical Data	Accuracy=98·5, Sensitivity=94·7%, Specificity=99·7%	10-fold cross validation
Baccour L. et al 2018 [155]	No	Diagnosis	Supervised	ATOVIC (hybrid multi-criteria decision making method)	N=7200	Clinical Data	Accuracy=92·7%, F-measure=95·3% (Hyper- vs Hypo- vs Control). Accuracy=99·81% (Hypo- vs Control)	Hold-out validation
Morejon et al. 2017 [156]	No	Diagnosis	Supervised	Java Agent Framework for Health Data Mining	.	Clinical Data	.	Hold-out validation
Temurtas et al. 2009 [157]	No	Diagnosis	Supervised	Probabilistic Neural Network	N=215 (n(normal)=150, n(hypo)=30, n(hyper)=35)	Clinical Data	Accuracy=94·81%	10-fold cross validation
Polat et al. 2007 [158]	No	Diagnosis	Supervised	Artificial Immune Recognition System with fuzzy weighted pre-processing	N=215 (n(normal)=150, n(hypo)=30, n(hyper)=35)	Clinical Data	Accuracy=85%	10-fold cross validation
Keles et al. 2008 [159]	No	Diagnosis	Supervised	Expert system for thyroid disease diagnosis with fuzzy rules	N=215 (n(normal)=150, n(hypo)=30, n(hyper)=35)	Clinical Data	Accuracy=95·33%	10-fold cross validation
**Autoimmune Liver Disease**								
Weiss J et al. 2015 [160]	No	Response to Treatment	Supervised	Boosted Forest	N=288	Clinical Trial Data	.	Hold-out validation
Singh et al. 2017 [161]	No	Disease Progression	Supervised	Kullback-Leibler Divergence-Least Squares Support Vector Machine	N=276	Clinical Data	Accuracy=90·94%	Hold-out validation
Eaton et al. 2018 [162]	No	Disease Progression	Supervised	Gradient Boosting	N=787	Clinical Data	Cross-validation: C-statistic=0·96 Independent test data: C-statistic=0·9	5-fold cross validation, independent test dataset

Paper	Multiple AIDs Studied	Prediction or Classification Task	ML Type	Machine Learning Method	Study Size (N)	Type of Data	Best Results (Metrics) Reported from validation or cross-validation, and where conducted, the test set.	Cross-Validation
Iwasawa et al. 2018 [163]	Yes	Diagnosis	Supervised	Random Forest	N= 64 (n(PSC)=24, n(UC)=16, n(HC)=24)	Microbiome Data	Genera: AUC=0·7423 (PSC vs HC), 0·8756 (PSC vs UC).<br>Species: AUC=0·8756 (PSC vs HC), 0·7626 (PSC vs UC)	10-fold cross-validation
Tsujitani et al. 2009 [164]	No	Survival Prediction	Supervised	Neural Network	N=312	Clinical Data	.	Delete-one cross-validation"
**Systemic Sclerosis**								
Zhu et al 2018 [165]	No	Diagnosis	Supervised and Unsupervised	Hierarchical Clustering and Support Vector Machine	N=37 (n(controls)=19, n(SSc)=18)	DNA and RNA of PBMC	Accuracy=100%, Sensitivity=100%, Specificity=100%	Hold-on-one-out cross-validation
Taroni et al. 2017 [166]	No	Response to treatment	Supervised	Support Vector Machine	.	Gene expression Data	.	.
Huang et al. 2015 [167]	No	Disease Progression	Supervised	Random Forest	N=119	Clinical and peripheral blood flow cytometry Data	Accuracy=95%	Hold-out cross-validation
Berks et al. 2014 [168]	No	Diagnosis	Supervised	Random Forest	N= 991 (n(train)=80 ; n(validate)=104 HC + 83 PR + 269 SSc; n(test)=104 HC + 83 PR + 268 SSc) images	Nailfold Capillaroscopy Data	Accuracy=93·6%, F-measure=71·5%, Precision=64·1%, Recall=80·9%	Hold-out validation, testing set
**Alopecia**								
Huang et al. 2013 [169]	Yes	Comorbidity analysis	Supervised	Natural Language Processing	N=3568 (n(AA)=2115) and N=416 (PAFS cohort)	Patient Data Repository	Validity=93·9%	Hold-out validation
**Vitiligo**								
Sheth et al. 2013 [170]	Yes	Comorbidity analysis	Supervised	Natural Language Processing	N=3280	Research Patient Data Repository	.	.

Supplementary References

[1] Briggs FBS, Yu JC, Davis MF, Jiangyang J, Fu S, Parrotta E, et al. Multiple sclerosis risk factors contribute to onset heterogeneity. Multiple Sclerosis and Related Disorders. 2019;28:11-6.
[2] Ahmadi A, Davoudi S, Daliri MR. Computer Aided Diagnosis System for multiple sclerosis disease based on phase to amplitude coupling in covert visual attention. Comput Methods Programs Biomed. 2019;169:9-18.
[3] Zhang H, Alberts E, Pongratz V, Mühlau M, Zimmer C, Wiestler B, et al. Predicting conversion from clinically isolated syndrome to multiple sclerosis–An imaging-based machine learning approach. NeuroImage: Clinical. 2019;21:101593.
[4] Zurita M, Montalba C, Labbé T, Cruz JP, Dalboni da Rocha J, Tejos C, et al. Characterization of relapsing-remitting multiple sclerosis patients using support vector machine classifications of functional and diffusion MRI data. NeuroImage: Clinical. 2018;20:724-30.
[5] Wang S-H, Tang C, Sun J, Yang J, Huang C, Phillips P, et al. Multiple Sclerosis Identification by 14-Layer Convolutional Neural Network With Batch Normalization, Dropout, and Stochastic Pooling. Frontiers in Neuroscience. 2018;12(818).
[6] Neeb H, Schenk J. Multivariate prediction of multiple sclerosis using robust quantitative MR-based image metrics. Zeitschrift für Medizinische Physik. 2018.
[7] Lotsch J, Schiffmann S, Schmitz K, Brunkhorst R, Lerch F, Ferreiros N, et al. Machine-learning based lipid mediator serum concentration patterns allow identification of multiple sclerosis patients with high accuracy. Scientific Reports. 2018;8(1):14884.
[8] Tacchella A, Romano S, Ferraldeschi M, Salvetti M, Zaccaria A, Crisanti A, et al. Collaboration between a human group and artificial intelligence can improve prediction of multiple sclerosis course: a proof-of-principle study. F1000Research. 2017;6:2172.
[9] Lopez C, Tucker S, Salameh T, Tucker C. An unsupervised machine learning method for discovering patient clusters based on genetic signatures. Journal of Biomedical Informatics. 2018;85:30-9.
[10] Supratak A, Datta G, Gafson AR, Nicholas R, Guo Y, Matthews PM. Remote Monitoring in the Home Validates Clinical Gait Measures for Multiple Sclerosis. Frontiers in Neurology. 2018;9(561).
[11] Saccà V, Sarica A, Novellino F, Barone S, Tallarico T, Filippelli E, et al. Evaluation of machine learning algorithms performance for the prediction of early multiple sclerosis from resting-state FMRI connectivity data. Brain Imaging and Behavior. 2018.
[12] Mowry EM, Hedström AK, Gianfrancesco MA, Shao X, Schaefer CA, Shen L, et al. Incorporating machine learning approaches to assess putative environmental risk factors for multiple sclerosis. Multiple Sclerosis and Related Disorders. 2018;24:135-41.
[13] Yoo Y, Tang LYW, Brosch T, Li DKB, Kolind S, Vavasour I, et al. Deep learning of joint myelin and T1w MRI features in normal-appearing brain tissue to distinguish between multiple sclerosis patients and healthy controls. NeuroImage: Clinical. 2018;17:169-78.
[14] Kiiski H, Jollans L, Donnchadha SÓ, Nolan H, Lonergan R, Kelly S, et al. Machine Learning EEG to Predict Cognitive Functioning and Processing Speed Over a 2-Year Period in Multiple Sclerosis Patients and Controls. Brain Topography. 2018;31(3):346-63.
[15] Fiorini S, Verri A, Tacchino A, Ponzio M, Brichetto G, Barla A. A machine learning pipeline for multiple sclerosis course detection from clinical scales and patient reported outcomes. Conf Proc IEEE Eng Med Biol Soc. 2015;2015:4443-46.
[16] Zhong J, Chen DQ, Nantes JC, Holmes SA, Hodaie M, Koski L. Combined structural and functional patterns discriminating upper limb motor disability in multiple sclerosis using multivariate approaches. Brain Imaging Behav. 2017;11(3):754-68.
[17] Lötsch J, Thrun M, Lerch F, Brunkhorst R, Schiffmann S, Thomas D, et al. Machine-Learned Data Structures of Lipid Marker Serum Concentrations in Multiple Sclerosis Patients Differ from Those in Healthy Subjects. International journal of molecular sciences. 2017;18(6):1217.

[18] Karaca Y, Zhang YD, Cattani C, Ayan U. The Differential Diagnosis of Multiple Sclerosis Using Convex Combination of Infinite Kernels. CNS Neurol Disord Drug Targets. 2017;16(1):36-43.
[19] Ostmeyer J, Christley S, Rounds WH, Toby I, Greenberg BM, Monson NL, et al. Statistical classifiers for diagnosing disease from immune repertoires: a case study using multiple sclerosis. BMC Bioinformatics. 2017;18(1):401.
[20] McGinnis RS, Mahadevan N, Moon Y, Seagers K, Sheth N, Wright JA, Jr., et al. A machine learning approach for gait speed estimation using skin-mounted wearable sensors: From healthy controls to individuals with multiple sclerosis. PLoS One. 2017;12(6):e0178366.
[21] Zhao Y, Healy BC, Rotstein D, Guttmann CRG, Bakshi R, Weiner HL, et al. Exploration of machine learning techniques in predicting multiple sclerosis disease course. PLOS ONE. 2017;12(4):e0174866.
[22] Ion-Mărgineanu A, Kocevar G, Stamile C, Sima DM, Durand-Dubief F, Van Huffel S, et al. Machine Learning Approach for Classifying Multiple Sclerosis Courses by Combining Clinical Data with Lesion Loads and Magnetic Resonance Metabolic Features. Frontiers in Neuroscience. 2017;11(398).
[23] Kocevar G, Stamile C, Hannoun S, Cotton F, Vukusic S, Durand-Dubief F, et al. Graph Theory-Based Brain Connectivity for Automatic Classification of Multiple Sclerosis Clinical Courses. Frontiers in Neuroscience. 2016;10(478).
[24] Kosa P, Ghazali D, Tanigawa M, Barbour C, Cortese I, Kelley W, et al. Development of a Sensitive Outcome for Economical Drug Screening for Progressive Multiple Sclerosis Treatment. Frontiers in Neurology. 2016;7(131).
[25] Baranzini SE, Madireddy LR, Cromer A, D'Antonio M, Lehr L, Beelke M, et al. Prognostic biomarkers of IFNb therapy in multiple sclerosis patients. Multiple sclerosis (Houndmills, Basingstoke, England). 2015;21(7):894-904.
[26] Wottschel V, Alexander DC, Kwok PP, Chard DT, Stromillo ML, De Stefano N, et al. Predicting outcome in clinically isolated syndrome using machine learning. Neuroimage Clin. 2015;7:281-7.
[27] Crimi A, Commowick O, Maarouf A, Ferre JC, Bannier E, Tourbah A, et al. Predictive value of imaging markers at multiple sclerosis disease onset based on gadolinium- and USPIO-enhanced MRI and machine learning. PLoS One. 2014;9(4):e93024.
[28] Sweeney EM, Vogelstein JT, Cuzzocreo JL, Calabresi PA, Reich DS, Crainiceanu CM, et al. A Comparison of Supervised Machine Learning Algorithms and Feature Vectors for MS Lesion Segmentation Using Multimodal Structural MRI. PLOS ONE. 2014;9(4):e95753.
[29] Taschler B, Ge T, Bendfeldt K, Müller-Lenke N, Johnson TD, Nichols TE, editors. Spatial Modeling of Multiple Sclerosis for Disease Subtype Prediction2014; Cham: Springer International Publishing.
[30] Alaqtash M, Sarkodie-Gyan T, Yu H, Fuentes O, Brower R, Abdelgawad A. Automatic classification of pathological gait patterns using ground reaction forces and machine learning algorithms. Conf Proc IEEE Eng Med Biol Soc. 2011;2011:453-7.
[31] Goldstein BA, Hubbard AE, Cutler A, Barcellos LF. An application of Random Forests to a genome-wide association dataset: methodological considerations & new findings. BMC genetics. 2010;11:49.
[32] Corvol JC, Pelletier D, Henry RG, Caillier SJ, Wang J, Pappas D, et al. Abrogation of T cell quiescence characterizes patients at high risk for multiple sclerosis after the initial neurological event. Proceedings of the National Academy of Sciences of the United States of America. 2008;105(33):11839-44.
[33] Briggs FB, Bartlett SE, Goldstein BA, Wang J, McCauley JL, Zuvich RL, et al. Evidence for CRHR1 in multiple sclerosis using supervised machine learning and meta-analysis in 12,566 individuals. Human molecular genetics. 2010;19(21):4286-95.
[34] Commowick O, Istace A, Kain M, Laurent B, Leray F, Simon M, et al. Objective Evaluation of Multiple Sclerosis Lesion Segmentation using a Data Management and Processing Infrastructure. Scientific Reports. 2018;8(1):13650.
[35] Ohanian D, Brown A, Sunnquist M, Furst J, Nicholson L, Klebek L, et al. Identifying Key Symptoms Differentiating Myalgic Encephalomyelitis and Chronic Fatigue Syndrome from Multiple Sclerosis. Neurology (E-Cronicon). 2016;4(2):41-5.

[36] Salem M, Cabezas M, Valverde S, Pareto D, Oliver A, Salvi J, et al. A supervised framework with intensity subtraction and deformation field features for the detection of new T2-w lesions in multiple sclerosis. NeuroImage: Clinical. 2018;17:607-15.
[37] Cabezas M, Oliver A, Valverde S, Beltran B, Freixenet J, Vilanova JC, et al. BOOST: A supervised approach for multiple sclerosis lesion segmentation. Journal of Neuroscience Methods. 2014;237:108-17.
[38] Zhang Y, Lu S, Zhou X, Yang M, Wu L, Liu B, et al. Comparison of machine learning methods for stationary wavelet entropy-based multiple sclerosis detection: decision tree, k-nearest neighbors, and support vector machine. Simulation. 2016;92(9):861-71.
[39] Birenbaum A, Greenspan H. Multi-view longitudinal CNN for multiple sclerosis lesion segmentation. Engineering Applications of Artificial Intelligence. 2017;65:111-8.
[40] Morrison C, Huckvale K, Corish B, Dorn J, Kontschieder P, O'Hara K, et al. Assessing Multiple Sclerosis With Kinect: Designing Computer Vision Systems for Real-World Use. Human-Computer Interaction. 2016;31(3/4):191-226.
[41] Liu J, Brodley CE, Healy BC, Chitnis T. Removing confounding factors via constraint-based clustering: An application to finding homogeneous groups of multiple sclerosis patients. Artificial Intelligence in Medicine. 2015;65(2):79-88.
[42] Chin CY, Hsieh SY, Tseng VS. EDram: Effective early disease risk assessment with matrix factorization on a large-scale medical database: A case study on rheumatoid arthritis. PLoS ONE. 2018;13 (11) (e0207579).
[43] Chocholova E, Bertok T, Jane E, Lorencova L, Holazova A, Belicka L, et al. Glycomics meets artificial intelligence - Potential of glycan analysis for identification of seropositive and seronegative rheumatoid arthritis patients revealed. Clinica Chimica Acta. 2018;481:49-55.
[44] Wu H, Cai L, Li D, Wang X, Zhao S, Zou F, et al. Metagenomics Biomarkers Selected for Prediction of Three Different Diseases in Chinese Population. BioMed Research International. 2018;2018 (2936257).
[45] Joo YB, Kim Y, Park Y, Kim K, Ryu JA, Lee S, et al. Biological function integrated prediction of severe radiographic progression in rheumatoid arthritis: A nested case control study. Arthritis and Rheumatology Conference: American College of Rheumatology/Association of Rheumatology Health Professionals Annual Scientific Meeting, ACR/ARHP. 2017;19(1):244.
[46] Andreu-Perez J, Garcia-Gancedo L, McKinnell J, Van der Drift A, Powell A, Hamy V, et al. Developing Fine-Grained Actigraphies for Rheumatoid Arthritis Patients from a Single Accelerometer Using Machine Learning. Sensors. 2017;17(9):2113.
[47] Orange DE, Agius P, DiCarlo EF, Robine N, Geiger H, Szymonifka J, et al. Identification of Three Rheumatoid Arthritis Disease Subtypes by Machine Learning Integration of Synovial Histologic Features and RNA Sequencing Data. Arthritis and Rheumatology. 2018;70(5):690-701.
[48] Ahmed U, Anwar A, Savage RS, Thornalley PJ, Rabbani N. Protein oxidation, nitration and glycation biomarkers for early-stage diagnosis of osteoarthritis of the knee and typing and progression of arthritic disease. Arthritis Res Ther. 2016;18(1):250.
[49] Miyoshi F, Honne K, Minota S, Okada M, Ogawa N, Mimura T. A novel method predicting clinical response using only background clinical data in RA patients before treatment with infliximab. Modern Rheumatology. 2016;26(6):813-6.
[50] Yeo L, Adlard N, Biehl M, Juarez M, Smallie T, Snow M, et al. Expression of chemokines CXCL4 and CXCL7 by synovial macrophages defines an early stage of rheumatoid arthritis. Annals of the Rheumatic Diseases. 2016;75(4):763-71.
[51] Zhou SM, Fernandez-Gutierrez F, Kennedy J, Cooksey R, Atkinson M, Denaxas S, et al. Defining disease phenotypes in primary care electronic health records by a machine learning approach: A case study in identifying rheumatoid arthritis. PLoS ONE. 2016;11 (5) (e0154515).
[52] Lin C, Karlson EW, Dligach D, Ramirez MP, Miller TA, Mo H, et al. Automatic identification of methotrexate-induced liver toxicity in patients with rheumatoid arthritis from the electronic medical record. J Am Med Inform Assoc. 2015;22(e1):e151-e61.
[53] Chen Y, Carroll RJ, Hinz ERM, Shah A, Eyler AE, Denny JC, et al. Applying active learning to high-throughput phenotyping algorithms for electronic health records data. J Am Med Inform Assoc. 2013;20:e253-e9.

[54] Lin C, Karlson EW, Canhao H, Miller TA, Dligach D, Chen PJ, et al. Automatic Prediction of Rheumatoid Arthritis Disease Activity from the Electronic Medical Records. PLoS ONE. 2013;8 (8) (e69932).
[55] Negi S, Juyal G, Senapati S, Prasad P, Gupta A, Singh S, et al. A genome-wide association study reveals ARL15, a novel non-HLA susceptibility gene for rheumatoid arthritis in North Indians. Arthritis and Rheumatism. 2013;65(12):3026-35.
[56] Pratt AG, Swan DC, Richardson S, Wilson G, Hilkens CMU, Young DA, et al. A CD4 T cell gene signature for early rheumatoid arthritis implicates interleukin 6-mediated STAT3 signalling, particularly in anti-citrullinated peptide antibody-negative disease. Annals of the Rheumatic Diseases. 2012;71(8):1374-81.
[57] Singh S, Kumar A, Panneerselvam K, Vennila JJ. Diagnosis of arthritis through fuzzy inference system. Journal of Medical Systems. 2012;36(3):1459-68.
[58] Kruppa J, Ziegler A, Konig IR. Risk estimation and risk prediction using machine-learning methods. Human Genetics. 2012;131(10):1639-54.
[59] Liu C, Ackerman HH, Carulli JP. A genome-wide screen of gene-gene interactions for rheumatoid arthritis susceptibility. Human Genetics. 2011;129(5):473-85.
[60] Nair SS, French RM, Laroche D, Thomas E. The Application of Machine Learning Algorithms to the Analysis of Electromyographic Patterns From Arthritic Patients. IEEE Trans Neural Syst Rehabil Eng. 2010;18(2):174-84.
[61] Briggs FBS, Ramsay PP, Madden E, Norris JM, Holers VM, Mikuls TR, et al. Supervised machine learning and logistic regression identifies novel epistatic risk factors with PTPN22 for rheumatoid arthritis. Genes and Immunity. 2010;11(3):199-208.
[62] Niu Q, Huang Z, Shi Y, Wang L, Pan X, Hu C. Specific serum protein biomarkers of rheumatoid arthritis detected by MALDI-TOF-MS combined with magnetic beads. International Immunology. 2010;22(7):611-8.
[63] Geurts P, Fillet M, de Seny D, Meuwis MA, Malaise M, Merville MP, et al. Proteomic mass spectra classification using decision tree based ensemble methods. Bioinformatics. 2005;21(14):3138-45.
[64] De Seny D, Fillet M, Meuwis MA, Geurts P, Lutteri L, Ribbens C, et al. Discovery of new rheumatoid arthritis biomarkers using the surface-enhanced laser desorption/ionization time-of-flight mass spectrometry proteinchip approach. Arthritis and Rheumatism. 2005;52(12):3801-12.
[65] Scheel AK, Netz UJ, Hermann KGA, Hielscher AH, Klose AD, Tresp V, et al. Laser Imaging Techniques for Follow-up Analysis of Joint Inflammation in Patients with Rheumatoid Arthritis. Medical Laser Application. 2003;18(3):198-205.
[66] Schwaighofe A, Tresp V, Mayer P, Krause A, Beuthan J, Rost H, et al. Classification of rheumatoid joint inflammation based on laser imaging. IEEE Transactions on Biomedical Engineering. 2003;50(3):375-82.
[67] Gronsbell J, Minnier J, Yu S, Liao K, Cai T. Automated Feature Selection of Predictors in Electronic Medical Records Data. Biometrics. 2018.
[68] Gossec L, Guyard F, Leroy D, Lafargue T, Seiler M, Jacquemin C, et al. Detection of flares by decrease in physical activity, collected using wearable activity trackers, in rheumatoid arthritis or axial spondyloarthritis: an application of Machine-Learning analyses in rheumatology. Arthritis Care Res (Hoboken). 2018;22:22.
[69] Lezcano-Valverde JM, Salazar F, Leon L, Toledano E, Jover JA, Fernandez-Gutierrez B, et al. Development and validation of a multivariate predictive model for rheumatoid arthritis mortality using a machine learning approach. Scientific Reports. 2017;7(1):10189.
[70] Gonzalez-Recio O, de Maturana EL, Vega AT, Engelman CD, Broman KW. Detecting single-nucleotide polymorphism by single-nucleotide polymorphism interactions in rheumatoid arthritis using a two-step approach with machine learning and a Bayesian threshold least absolute shrinkage and selection operator (LASSO) model. BMC Proc. 2009;3 Suppl 7:S63.
[71] Heard BJ, Rosvold JM, Fritzler MJ, El-Gabalawy H, Wiley JP, Krawetz RJ. A computational method to differentiate normal individuals, osteoarthritis and rheumatoid arthritis patients using serum biomarkers. J R Soc Interface. 2014;11(97):20140428.
[72] Gronsbell JL, Cai T. Semi‐supervised approaches to efficient evaluation of model prediction performance. Journal of the Royal Statistical Society: Series B (Statistical Methodology). 2018;80(3):579-94.

[73] Van Looy S, Vander Cruyssen B, Meeus J, Wyns B, Westhovens R, Durez P, et al. Prediction of dose escalation for rheumatoid arthritis patients under infliximab treatment. Engineering Applications of Artificial Intelligence. 2006;19(7):819-28.
[74] Wyns B, Boullart L, Sette S, Baeten D, Hoffman I, De Keyser F. Prediction of arthritis using a modified Kohonen mapping and case based reasoning. Engineering Applications of Artificial Intelligence. 2004;17(2):205.
[75] Waljee AK, Lipson R, Wiitala WL, Zhang Y, Liu B, Zhu J, et al. Predicting Hospitalization and Outpatient Corticosteroid Use in Inflammatory Bowel Disease Patients Using Machine Learning. Inflammatory Bowel Diseases. 2018;24(1):45-53.
[76] Mossotto E, Ashton JJ, Coelho T, Beattie RM, MacArthur BD, Ennis S. Classification of Paediatric Inflammatory Bowel Disease using Machine Learning. Scientific reports. 2017;7(1):2427.
[77] Maeda Y, Kudo SE, Mori Y, Misawa M, Ogata N, Sasanuma S, et al. Fully automated diagnostic system with artificial intelligence using endocytoscopy to identify the presence of histologic inflammation associated with ulcerative colitis (with video). Gastrointestinal Endoscopy. 2018;89(2):408-15.
[78] Douglas GM, Hansen R, Jones CM, Dunn KA, Comeau AM, Bielawski JP, et al. Multi-omics differentially classify disease state and treatment outcome in pediatric Crohn's disease. Microbiome. 2018;6 (1) (13).
[79] Jain S, Kedia S, Sethi T, Bopanna S, Yadav D, Goyal S, et al. Predictors of long-term outcomes in patients with acute severe ulcerative colitis: A northern Indian cohort study. Gastroenterology. 2017;152 (5 Supplement 1):S372.
[80] Waljee AK, Sauder K, Patel A, Segar S, Liu B, Zhang Y, et al. Machine learning algorithms for objective remission and clinical outcomes with thiopurines. Journal of Crohn's and Colitis. 2017;11(7):801-10.
[81] Isakov O, Dotan I, Ben-Shachar S. Machine Learning-Based Gene Prioritization Identifies Novel Candidate Risk Genes for Inflammatory Bowel Disease. Inflammatory Bowel Diseases. 2017;23(9):1516-23.
[82] Kang T, Ding W, Zhang L, Ziemek D, Zarringhalam K. A biological network-based regularized artificial neural network model for robust phenotype prediction from gene expression data. BMC bioinformatics. 2017;18(1):565.
[83] Waljee AK, Liu B, Sauder K, Zhu J, Govani SM, Stidham RW, et al. Predicting corticosteroid-free endoscopic remission with vedolizumab in ulcerative colitis. Alimentary Pharmacology and Therapeutics. 2018;47(6):763-72.
[84] Pal LR, Kundu K, Yin Y, Moult J. CAGI4 Crohn's exome challenge: Marker SNP versus exome variant models for assigning risk of Crohn disease. Human Mutation. 2017;38(9):1225-34.
[85] Eck A, Zintgraf LM, de Groot EFJ, de Meij TGJ, Cohen TS, Savelkoul PHM, et al. Interpretation of microbiota-based diagnostics by explaining individual classifier decisions. BMC bioinformatics. 2017;18(1):441.
[86] Menti E, Lanera C, Lorenzoni G, Giachino DF, Marchi M, Gregori D, et al. Bayesian Machine Learning Techniques for revealing complex interactions among genetic and clinical factors in association with extra-intestinal Manifestations in IBD patients. Amia 2016;Annual Symposium proceedings. AMIA Symposium. 2016:884-93.
[87] Hubenthal M, Hemmrich-Stanisak G, Degenhardt F, Szymczak S, Du Z, Elsharawy A, et al. Sparse modeling reveals miRNA signatures for diagnostics of inflammatory bowel disease. PLoS ONE. 2015;10 (10) (e140155).
[88] Niehaus KE, Uhlig HH, Clifton DA. Phenotypic characterisation of Crohn's disease severity. Conference proceedings : . 2015;Annual International Conference of the IEEE Engineering in Medicine and Biology Society. IEEE Engineering in Medicine and Biology Society. Annual Conference. 2015:7023-6.
[89] Wei Z, Wang W, Bradfield J, Li J, Cardinale C, Frackelton E, et al. Large sample size, wide variant spectrum, and advanced machine-learning technique boost risk prediction for inflammatory bowel disease. American Journal of Human Genetics. 2013;92(6):1008-12.
[90] Cui H, Zhang X. Alignment-free supervised classification of metagenomes by recursive SVM. BMC Genomics. 2013;14 (1) (641).
[91] Waljee AK, Joyce JC, Wang S, Saxena A, Hart M, Zhu J, et al. Algorithms Outperform Metabolite Tests in Predicting Response of Patients With Inflammatory Bowel Disease to Thiopurines. Clinical Gastroenterology and Hepatology. 2010;8(2):143-50.

[92] Firouzi F, Rashidi M, Hashemi S, Kangavari M, Bahari A, Daryani NE, et al. A decision tree-based approach for determining low bone mineral density in inflammatory bowel disease using WEKA software. European Journal of Gastroenterology and Hepatology. 2007;19(12):1075-81.
[93] Ozawa T, Ishihara S, Fujishiro M, Saito H, Kumagai Y, Shichijo S, et al. Novel Computer-assisted Diagnosis System for Endoscopic Disease Activity in Patients with Ulcerative Colitis. Gastrointestinal Endoscopy. 2018.
[94] Reddy BK, Delen D, Agrawal RK. Predicting and explaining inflammation in Crohn's disease patients using predictive analytics methods and electronic medical record data. Health Inform J. 2018:1460458217751015.
[95] Forbes JD, Chen CY, Knox NC, Marrie RA, El-Gabalawy H, de Kievit T, et al. A comparative study of the gut microbiota in immune-mediated inflammatory diseases-does a common dysbiosis exist? Microbiome. 2018;6(1):221.
[96] Doherty MK, Ding T, Koumpouras C, Telesco SE, Monast C, Das A, et al. Fecal Microbiota Signatures Are Associated with Response to Ustekinumab Therapy among Crohn's Disease Patients. mBio. 2018;9(2):e02120-17.
[97] Han L, Maciejewski M, Gordon W, Afzelius L, Brockel C, Snapper SB, et al. A probabilistic pathway score (PROPS) for classification with applications to inflammatory bowel disease. Bioinformatics. 2018;34(6):985-93.
[98] Daneshjou R, Wang Y, Bromberg Y, Bovo S, Martelli PL, Babbi G, et al. Working toward precision medicine: Predicting phenotypes from exomes in the Critical Assessment of Genome Interpretation (CAGI) challenges. Human Mutation. 2017;38(9):1182-92.
[99] Giollo M, Jones DT, Carraro M, Leonardi E, Ferrari C, Tosatto SCE. Crohn disease risk prediction-Best practices and pitfalls with exome data. Human Mutation. 2017;38(9):1193-200.
[100] Yu S, Chakrabortty A, Liao KP, Cai T, Ananthakrishnan AN, Gainer VS, et al. Surrogate-assisted feature extraction for high-throughput phenotyping. J Am Med Inform Assoc. 2017;24(e1):e143-e9.
[101] Wisittipanit N, Rangwala H, Sikaroodi M, Keshavarzian A, Mutlu EA, Gillevet P. Classification methods for the analysis of LH-PCR data associated with inflammatory bowel disease patients. Int J Bioinform Res Appl. 2015;11(2):111-29.
[102] Ahmed S, Dey N, Ashour A, Sifaki-Pistolla D, Bălas-Timar D, Balas V, et al. Effect of fuzzy partitioning in Crohn's disease classification: a neuro-fuzzy-based approach. Med Biol Eng Comput. 2017;55(1):101-15.
[103] Mahapatra D, Vos FM, Buhmann JM. Active learning based segmentation of Crohns disease from abdominal MRI. Comput Methods Programs Biomed. 2016;128:75-85.
[104] Mahapatra D. Combining multiple expert annotations using semi-supervised learning and graph cuts for medical image segmentation. Computer Vision & Image Understanding. 2016;151:114-23.
[105] Stawiski K, Pietrzak I, Mlynarski W, Fendler W, Szadkowska A. NIRCa: An artificial neural network-based insulin resistance calculator. Pediatric Diabetes. 2018;19(2):231-5.
[106] Ben Ali J, Hamdi T, Fnaiech N, Di Costanzo V, Fnaiech F, Ginoux JM. Continuous blood glucose level prediction of Type 1 Diabetes based on Artificial Neural Network. Biocybernetics and Biomedical Engineering. 2018;38(4):828-40.
[107] Perez-Gandia C, Garcia-Saez G, Subias D, Rodriguez-Herrero A, Gomez EJ, Rigla M, et al. Decision Support in Diabetes Care: The Challenge of Supporting Patients in Their Daily Living Using a Mobile Glucose Predictor. Journal of Diabetes Science and Technology. 2018;12(2):243-50.
[108] Maulucci G, Cordelli E, Rizzi A, De Leva F, Papi M, Ciasca G, et al. Phase separation of the plasma membrane in human red blood cells as a potential tool for diagnosis and progression monitoring of type 1 diabetes mellitus. PLoS ONE. 2017;12 (9) (e0184109).
[109] Siegel AP, Daneshkhah A, Hardin DS, Shrestha S, Varahramyan K, Agarwal M. Analyzing breath samples of hypoglycemic events in type 1 diabetes patients: Towards developing an alternative to diabetes alert dogs. Journal of Breath Research. 2017;11 (2) (026007).

[110] Zhao LP, Bolouri H, Zhao M, Geraghty DE, Lernmark A. An Object-Oriented Regression for Building Disease Predictive Models with Multiallelic HLA Genes. Genetic Epidemiology. 2016;40(4):315-32.
[111] Georga EI, Protopappas VC, Polyzos D, Fotiadis DI. Online prediction of glucose concentration in type 1 diabetes using extreme learning machines. Conference proceedings : . 2015;Annual International Conference of the IEEE Engineering in Medicine and Biology Society. IEEE Engineering in Medicine and Biology Society. Annual Conference. 2015:3262-5.
[112] Georga EI, Protopappas VC, Ardigo D, Polyzos D, Fotiadis DI. A Glucose Model Based on Support Vector Regression for the Prediction of Hypoglycemic Events Under Free-Living Conditions. Diabetes Technology and Therapeutics. 2013;15(8):634-43.
[113] Marling CR, Struble NW, Bunescu RC, Shubrook JH, Schwartz FL. A consensus-perceived glycemic variability metric. Journal of Diabetes Science and Technology. 2013;7 (4):871-79.
[114] Nguyen C, Varney MD, Harrison LC, Morahan G. Definition of high-risk type 1 diabetes HLA-DR and HLA-DQ types using only three single nucleotide polymorphisms. Diabetes. 2013;62(6):2135-40.
[115] Wei Z, Wang K, Qu HQ, Zhang H, Bradfield J, Kim C, et al. From disease association to risk assessment: An optimistic view from genome-wide association studies on type 1 diabetes. PLoS Genetics. 2009;5 (10)(e1000678).
[116] Jensen MH, Mahmoudi Z, Christensen TF, Tarnow L, Seto E, Johansen MD, et al. Evaluation of an Algorithm for Retrospective Hypoglycemia Detection Using Professional Continuous Glucose Monitoring Data. J Diabetes Sci Technol. 2014;8(1):117-22.
[117] Schwartz FL, Shubrook JH, Marling CR. Use of case-based reasoning to enhance intensive management of patients on insulin pump therapy. J Diabetes Sci Technol. 2008;2(4):603-11.
[118] Cordelli E, Maulucci G, De Spirito M, Rizzi A, Pitocco D, Soda P. A decision support system for type 1 diabetes mellitus diagnostics based on dual channel analysis of red blood cell membrane fluidity. Comput Methods Programs Biomed. 2018;162:263-71.
[119] Sampath S, Tkachenko P, Renard E, Pereverzev SV. Glycemic Control Indices and Their Aggregation in the Prediction of Nocturnal Hypoglycemia From Intermittent Blood Glucose Measurements. J Diabetes Sci Technol. 2016;10(6):1245-50.
[120] Georga EI, Protopappas VC, Polyzos D, Fotiadis DI. Evaluation of short-term predictors of glucose concentration in type 1 diabetes combining feature ranking with regression models. Med Biol Eng Comput. 2015;53(12):1305-18.
[121] Ling SH, San PP, Nguyen HT. Non-invasive hypoglycemia monitoring system using extreme learning machine for Type 1 diabetes. ISA Transactions. 2016;64:440-6.
[122] Ceccarelli F, Sciandrone M, Perricone C, Galvan G, Cipriano E, Galligari A, et al. Biomarkers of erosive arthritis in systemic lupus erythematosus: Application of machine learning models. PLoS ONE. 2018;13 (12) (e0207926).
[123] Turner CA, Jacobs AD, Marques CK, Oates JC, Kamen DL, Anderson PE, et al. Word2Vec inversion and traditional text classifiers for phenotyping lupus. BMC medical informatics and decision making. 2017;17(1):126.
[124] Ceccarelli F, Sciandrone M, Perricone C, Galvan G, Morelli F, Vicente LN, et al. Prediction of chronic damage in systemic lupus erythematosus by using machine-learning models. PLoS ONE. 2017;12 (3)(e0174200).
[125] Kan H, Nagar S, Patel J, Wallace DJ, Molta C, Chang DJ. Longitudinal Treatment Patterns and Associated Outcomes in Patients with Newly Diagnosed Systemic Lupus Erythematosus. Clinical Therapeutics. 2016;38(3):610-24.
[126] Wolf BJ, Spainhour JC, Arthur JM, Janech MG, Petri M, Oates JC. Development of Biomarker Models to Predict Outcomes in Lupus Nephritis. Arthritis and Rheumatology. 2016;68(8):1955-63.
[127] Guy RT, Santago P, Langefeld CD. Bootstrap Aggregating of Alternating Decision Trees to Detect Sets of SNPs That Associate With Disease. Genetic Epidemiology. 2012;36(2):99-106.

[128] Tang H, Poynton MR, Hurdle JF, Baird BC, Koford JK, Goldfarb-Rumyantzev AS. Predicting three-year kidney graft survival in recipients with systemic lupus erythematosus. ASAIO journal (American Society for Artificial Internal Organs : 1992). 2011;57(4):300-9.
[129] Armañanzas R, Calvo B, Inza I, López-Hoyos M, Martínez-Taboada V, Ucar E, et al. Microarray Analysis of Autoimmune Diseases by Machine Learning Procedures. IEEE Transactions on Information Technology in Biomedicine. 2009;13(3):341-50.
[130] Huang Z, Shi Y, Cai B, Wang L, Wu Y, Ying B, et al. MALDI-TOF MS combined with magnetic beads for detecting serum protein biomarkers and establishment of boosting decision tree model for diagnosis of systemic lupus erythematosus. Rheumatology. 2009;48(6):626-31.
[131] Murray SG, Avati A, Schmajuk G, Yazdany J. Automated and flexible identification of complex disease: building a model for systemic lupus erythematosus using noisy labeling. J Am Med Inform Assoc. 2018;26(1):61-5.
[132] Reddy BK, Delen D. Predicting hospital readmission for lupus patients: An RNN-LSTM-based deep-learning methodology. Comput Biol Med. 2018;101:199-209.
[133] Tang Y, Zhang W, Zhu M, Zheng L, Xie L, Yao Z, et al. Lupus nephritis pathology prediction with clinical indices. Scientific Reports. 2018;8(1):10231.
[134] Scully M, Anderson B, Lane T, Gasparovic C, Magnotta V, Sibbitt W, et al. An Automated Method for Segmenting White Matter Lesions through Multi-Level Morphometric Feature Classification with Application to Lupus. Front Hum Neurosci. 2010;4:27.
[135] Davis NA, Lareau CA, White BC, Pandey A, Wiley G, Montgomery CG, et al. Encore: Genetic Association Interaction Network centrality pipeline and application to SLE exome data. Genetic Epidemiology. 2013;37(6):614-21.
[136] Wang Y, Li Y, Pu W, Wen K, Shugart YY, Xiong M, et al. Random Bits Forest: a Strong Classifier/Regressor for Big Data. Scientific reports. 2016;6:30086.
[137] George Y, Aldeen M, Garnavi R. Psoriasis image representation using patch-based dictionary learning for erythema severity scoring. Computerized Medical Imaging & Graphics. 2018;66:44-55.
[138] Shrivastava VK, Londhe ND, Sonawane RS, Suri JS. A novel and robust Bayesian approach for segmentation of psoriasis lesions and its risk stratification. Comput Methods Programs Biomed. 2017;150:9-22.
[139] Shrivastava VK, Londhe ND, Sonawane RS, Suri JS. Computer-aided diagnosis of psoriasis skin images with HOS, texture and color features: A first comparative study of its kind. Comput Methods Programs Biomed. 2016;126:98-109.
[140] Shrivastava VK, Londhe ND, Sonawane RS, Suri JS. A novel approach to multiclass psoriasis disease risk stratification: Machine learning paradigm. Biomedical Signal Processing and Control. 2016;28:27-40.
[141] Shrivastava VK, Londhe ND, Sonawane RS, Suri JS. Exploring the color feature power for psoriasis risk stratification and classification: A data mining paradigm. Computers in Biology and Medicine. 2015;65:54-68.
[142] Cowen EW, Liu CW, Steinberg SM, Kang S, Vonderheid EC, Kwak HS, et al. Differentiation of tumour-stage mycosis fungoides, psoriasis vulgaris and normal controls in a pilot study using serum proteomic analysis. British Journal of Dermatology. 2007;157(5):946-53.
[143] Raina A, Hennessy R, Rains M, Allred J, Hirshburg JM, Diven DG, et al. Objective measurement of erythema in psoriasis using digital color photography with color calibration. Skin Res Technol. 2016;22(3):375-80.
[144] Shrivastava VK, Londhe ND, Sonawane RS, Suri JS. Reliable and accurate psoriasis disease classification in dermatology images using comprehensive feature space in machine learning paradigm. Expert Systems with Applications. 2015;42(15/16):6184-95.
[145] Shrivastava VK, Londhe ND, Sonawane RS, Suri JS. Reliability analysis of psoriasis decision support system in principal component analysis framework. Data & Knowledge Engineering. 2016;106:1-17.
[146] Patrick MT, Stuart PE, Raja K, Gudjonsson JE, Tejasvi T, Yang J, et al. Genetic signature to provide robust risk assessment of psoriatic arthritis development in psoriasis patients. Nature Communications. 2018;9 (1) (4178).
[147] Hujoel IA, Murphree DH, Van Dyke CT, Choung RS, Sharma A, Murray JA, et al. Machine Learning in Detection of Undiagnosed Celiac Disease. Clinical Gastroenterology and Hepatology. 2018;16(8):1354-5.e1.

[148] Arasaradnam RP, Westenbrink E, McFarlane MJ, Harbord R, Chambers S, O'Connell N, et al. Differentiating coeliac disease from irritable bowel syndrome by urinary volatile organic compound analysis - A pilot study. PLoS ONE. 2014;9 (10) (e107312).
[149] Tenorio JM, Hummel AD, Cohrs FM, Sdepanian VL, Pisa IT, De Fatima Marin H. Artificial intelligence techniques applied to the development of a decision-support system for diagnosing celiac disease. International Journal of Medical Informatics. 2011;80(11):793-802.
[150] Choung RS, Rostamkolaei SK, Ju JM, Marietta EV, Van Dyke CT, Rajasekaran JJ, et al. Synthetic Neoepitopes of the Transglutaminase-Deamidated Gliadin Complex as Biomarkers for Diagnosing and Monitoring Celiac Disease. Gastroenterology. 2019;156(3):582-91.e1.
[151] Chen W, Huang Y, Boyle B, Lin S. The utility of including pathology reports in improving the computational identification of patients. J Pathol Inform. 2016;7:46.
[152] Ludvigsson JF, Pathak J, Murphy S, Durski M, Kirsch PS, Chute CG, et al. Use of computerized algorithm to identify individuals in need of testing for celiac disease. J Am Med Inform Assoc. 2013;20(e2):e306-10.
[153] Amirkhani A, Mosavi MR, Mohammadi K, Papageorgiou EI. A novel hybrid method based on fuzzy cognitive maps and fuzzy clustering algorithms for grading celiac disease. Neural Computing & Applications. 2018;30(5):1573-88.
[154] Ahmad W, Ahmad A, Lu C, Khoso BA, Huang L. A novel hybrid decision support system for thyroid disease forecasting. Soft Computing - A Fusion of Foundations, Methodologies & Applications. 2018;22(16):5377-83.
[155] Baccour L. Amended fused TOPSIS-VIKOR for classification (ATOVIC) applied to some UCI data sets. Expert Systems with Applications. 2018;99:115-25.
[156] Morejón R, Viana M, Lucena C. An Approach to Generate Software Agents for Health Data Mining. International Journal of Software Engineering & Knowledge Engineering. 2017;27(9/10):1579-89.
[157] Temurtas F. A comparative study on thyroid disease diagnosis using neural networks. Expert Systems with Applications. 2009;36(1):944-9.
[158] Polat K, Şahan S, Güneş S. A novel hybrid method based on artificial immune recognition system (AIRS) with fuzzy weighted pre-processing for thyroid disease diagnosis. Expert Systems with Applications. 2007;32(4):1141-7.
[159] Keleş A, Keleş A. ESTDD: Expert system for thyroid diseases diagnosis. Expert Systems with Applications. 2008;34(1):242-6.
[160] Weiss J, Kuusisto F, Boyd K, Liu J, Page D. Machine Learning for Treatment Assignment: Improving Individualized Risk Attribution. Amia 2015;Annual Symposium proceedings. AMIA Symposium. 2015:1306-15.
[161] Singh A, Pandey B. A KLD-LSSVM based computational method applied for feature ranking and classification of primary biliary cirrhosis stages. International Journal of Computational Biology and Drug Design. 2017;10(1):24-38.
[162] Eaton JE, Vesterhus M, McCauley BM, Atkinson EJ, Schlicht EM, Juran BD, et al. Primary Sclerosing Cholangitis Risk Estimate Tool (PREsTo) Predicts Outcomes in PSC: A Derivation & Validation Study Using Machine Learning. Hepatology. 2018.
[163] Iwasawa K, Suda W, Tsunoda T, Oikawa-Kawamoto M, Umetsu S, Takayasu L, et al. Dysbiosis of the salivary microbiota in pediatric-onset primary sclerosing cholangitis and its potential as a biomarker. Scientific Reports. 2018;8(1):5480.
[164] Tsujitani M, Sakon M. Analysis of Survival Data Having Time-Dependent Covariates. IEEE Transactions on Neural Networks. 2009;20(3):389-94.
[165] Zhu H, Zhu C, Mi W, Chen T, Zhao H, Zuo X, et al. Integration of Genome-Wide DNA Methylation and Transcription Uncovered Aberrant Methylation-Regulated Genes and Pathways in the Peripheral Blood Mononuclear Cells of Systemic Sclerosis. International Journal of Rheumatology. 2018;2018 (no pagination)(7342472).
[166] Taroni JN, Martyanov V, Mahoney JM, Whitfield ML. A Functional Genomic Meta-Analysis of Clinical Trials in Systemic Sclerosis: Toward Precision Medicine and Combination Therapy. Journal of Investigative Dermatology. 2017;137(5):1033-41.
[167] Huang H, Fava A, Guhr T, Cimbro R, Rosen A, Boin F, et al. A methodology for exploring biomarker--phenotype associations: application to flow cytometry data and systemic sclerosis clinical manifestations. BMC bioinformatics. 2015;16:293.
[168] Berks M, Tresadern P, Dinsdale G, Murray A, Moore T, Herrick A, et al. An automated system for detecting and measuring nailfold capillaries. Medical image computing and computer-assisted intervention : MICCAI 2014;International Conference on Medical Image Computing and Computer-Assisted Intervention. 17(Pt 1):658-65.

[169] Huang KP, Mullangi S, Guo Y, Qureshi AA. Autoimmune, atopic, and mental health comorbid conditions associated with alopecia areata in the United States.[Erratum appears in JAMA Dermatol. 2014 Jun;150(6):674]. JAMA Dermatology. 2013;149(7):789-94.
[170] Sheth VM, Guo Y, Qureshi AA. Comorbidities associated with vitiligo: A ten-year retrospective study. Dermatology. 2013;227(4):311-5.
